# Factors Influencing Consumption Behaviour towards Aquatic Food among Asian Consumers: A Systematic Scoping Review

**DOI:** 10.3390/foods11244043

**Published:** 2022-12-14

**Authors:** Mausam Budhathoki, Danny Campbell, Ben Belton, Richard Newton, Saihong Li, Wenbo Zhang, David Little

**Affiliations:** 1 Institute of Aquaculture, University of Stirling, Stirling FK9 4LA, UK; 2 Division of Economics, School of Management, University of Stirling, Stirling FK9 5AE, UK; 3 Department of Agricultural, Food, and Resource Economics, Michigan State University, East Lansing, MI 48824, USA; 4 WorldFish, Jalan Batu Maung, Pulau Pinang 11960, Malaysia; 5 Faculty of Arts and Humanities, University of Stirling, Stirling FK9 4LA, UK; 6 National Demonstration Center for Experimental Fisheries Science Education, Shanghai Ocean University, Shanghai 201308, China

**Keywords:** fish consumption, consumer behaviour, seafood preference, fisheries, aquaculture, Asia

## Abstract

Asia accounts for over 70% of total global aquatic food consumption, but aquatic food consumption behaviours and attitudes among Asian consumers are poorly documented and understood. This paper synthesises literature on factors influencing aquatic food consumption behaviour in Asia and the potential to support transitions toward more sustainable food consumption patterns. We identified 113 studies for inclusion in a scoping review, and identified five clusters of publications: (1) product attributes, availability, and accessibility (24% of publications); (2) willingness to pay for aquatic foods (25%); (3) psychosocial factors (e.g., attitudes and subjective norms) (17%); (4) sociodemographic and lifestyle factors (21%); and (5) miscellaneous factors, including food safety and social status (13%). This study indicates that multiple interacting factors influence aquatic food consumption behaviours among Asian consumers, among which price is central. Knowledge of, and attitudes toward, the perceived quality and safety of aquatic foods were identified as important but were mediated by household characteristics. Sustainable production practices, country of origin, and ecolabels were found to be less influential on consumption behaviour. We found that improving consumers’ knowledge and attitudes about the quality and safety of aquatic foods might positively influence aquatic food consumption behaviour. Future multidisciplinary research is required to better understand interactions among the multiple factors that influence Asian consumers’ aquatic food consumption behaviour.

## 1. Introduction

The landmark EAT-Lancet Commission issued recommendations for responsible food consumption within planetary boundaries [1], but aquatic food was considered as a single commodity group [2], and various aspects of food systems such as affordability and cultural and demographic variations in nutritional sufficiency were not considered [3,4]. Understanding of the diversity and impact of aquatic food consumption on broader food security and sustainability has been limited and has often ignored perceptions of seafood as a dietary component and any associated behaviours. The Blue Food Assessment has established a baseline to build an understanding of the role of aquatic food consumption in global food systems [5,6,7,8]. Researchers have found that the inclusion of aquatic foods in the diet can provide more sustainable food options than terrestrial animal production [9,10,11,12] and has the potential to meet the characteristics of a sustainable diet [10,13,14]. Further, several previous studies have indicated that the consumption of aquatic foods is critical to food and nutrition security for many vulnerable groups [15,16,17].

Eating behaviour is a complex phenomenon determined by several interacting factors that go far beyond its functional roles in mitigating hunger or providing nutrition and often include personal and socio-cultural factors [18,19,20,21,22,23]. Some authors [24] suggest that the food choice decisions that impact food consumption behaviour are frequent, multifaceted, situational, dynamic, and complex; thus, it is difficult to capture the full complexity of eating behaviour using any given theory, framework, or model. To date, known factors that affect aquatic food consumption behaviour include availability, price, self-efficacy, convenience, habit, health and nutrition beliefs, sensory perception, country of origin, production method, preservation method, product innovation, packaging, eco-labels, safety, culture, and religion, as well as socio-demographic characteristics [25,26,27,28,29,30,31,32].

According to the Food and Agriculture Organization (FAO), Asian consumers enjoy high consumption levels of aquatic food (24.6 kg/capita/year), and in 2019, Asian consumers consumed over 70% of total global aquatic food [33]. However, there is vast heterogeneity in aquatic food consumption among countries within the Asian region [33,34], areas within countries (for instance, rural versus urban) [35], between households, and even at an intra-household level [36]. This might be due to various reasons including differences in economic development [7], technological innovation [15], infrastructure (cold-chain storage) and distribution channels, and availability [37,38,39], as well as consumers’ preferences [40,41]. Further, compared to six geographic regions of the world, the level of urbanization in Asia more than doubled from 17.5% in 1950 to 49.9% in 2018, and Asia experienced the highest average annual rate of urbanisation of 1.6% from 1990 to 2018 [42]. Thus, in line with ‘Bennett’s Law’, consumers in Asia, especially those residing in urban areas with rising income, reflect a desire for dietary diversity to significantly increase the consumption of non-grain products (fruits, vegetables, meat, dairy, and fish) compared with grains and other starchy staples [7,43,44]. Thus, in a market driven by demand [45], a better understanding of consumption behaviour towards aquatic food is foremost in developing more effective marketing and policy strategies.

Preferences, choices, and habits occupy a central role in consumption behaviour towards aquatic food. Cairns [18] identified that marketing efforts targeted toward food choices in general could shift preferences and result in changed dietary norms in food and drink categories (at the population level) and in the cultural values underpinning food behaviours. However, studies investigating consumption behaviour towards aquatic food among Asian consumers remained highly fragmented and disorganized as nations in the Asian region have their own pre-existing national and local food cultures and traditions. Thus, the available studies vary greatly in terms of objectives, methodology, sampling technique, and their focus on differences in socio-demographic and lifestyle characteristics. In addition, compared to other food categories, aquatic foods are highly heterogeneous in terms of production method, type/species, origin, and processing form. For instance, more than 2500 species or species groups of fish, invertebrates, algae, and aquatic plants are currently used for human consumption [6,11]. Further, unlike many Western consumers, Asian consumers often prefer to buy whole and/or live fish, whether in wet markets, restaurants, supermarkets, or online. From a marketing perspective, a better understanding of factors influencing consumption behaviour towards aquatic food is crucial, especially when diverse aquatic food consumption patterns and habits are involved.

So far, no review has been conducted that is aimed at establishing insights into consumption behaviour towards aquatic food in Asia, or retrieving and considering any factors associated with consumption behaviour. Thus, there is a challenge in understanding the key factors influencing consumers’ aquatic food consumption behaviour in the wider Asian context. In order to fill this gap, this paper identifies and examines the main findings of research on consumption behaviour towards aquatic foods among consumers in East Asia, Southeast Asia, and South Asia through a scoping literature review. In this study, aquatic foods include the full range of aquatic animals, plants, and microorganisms that can be eaten and that originate in bodies of water.

## 2. Materials and Methods

Scoping reviews examine emerging evidence and identify research gaps in the existing literature while maintaining the same methodological rigour as systematic reviews [46]. Previous studies have pointed out that a scoping review is particularly beneficial when formulating more precise questions than can be addressed by a systematic review [47,48]. Further, Peterson et al. [49] argue that a scoping review is particularly beneficial for complex and interdisciplinary areas of the literature, such as food consumption behaviour. The planning, development, and reporting of this scoping review have been informed by previous literature on a methodological framework for scoping reviews [46,50,51,52]. We strictly followed the PRISMA scoping review procedure in the paper.

### 2.1. Identification and Selection

A range of electronic databases was employed to locate records published between 1 January 2010 and 31 January 2022 (83% of the publications included in the final sample were published after 2015), written in the English language and targeting three regions of Asia: East Asia, Southeast Asia, and South Asia. Search databases included the Web of Science, Scopus, and Google Scholar. An initial search was conducted in January 2022 that included all records with a title or abstract containing the following search string: (aquatic OR seafood OR fish OR shellfish) AND (consum* OR eat* OR intake) AND (behav* OR intention OR choice* OR attribute OR preference OR attitud* OR habit OR buy* OR purchas* OR willing* OR perception) AND (Asia OR “South Asia” OR “Southeast Asia” OR “Northeast Asia” OR “East Asia” OR China OR Japan OR “South Korea” OR “North Korea” OR Taiwan OR “Hong Kong” OR Mongolia OR Macao OR Brunei OR Burma OR Myanmar OR Cambodia OR Timor-Leste OR Indonesia OR Laos OR Malaysia OR Philippines OR Singapore OR Thailand OR Vietnam OR India OR Bangladesh OR Nepal OR Maldives OR “Sri Lanka” OR Bhutan OR Pakistan OR Afghanistan). The search string was adapted to the syntax of each database.

For this purpose, aquatic foods include the full range of aquatic animals, plants, and microorganisms that can be eaten and that originate in bodies of water. Inclusion criteria include all types of records, including reviews, reports, and research (both qualitative and quantitative) published in the English language, available as full texts, and conducted among consumers of East Asia, Southeast Asia, and South Asia. Exclusion criteria include search terms in a different context to the research question (for instance, analytical sensory analysis of aquatic foods, logistics of aquatic foods, and business modelling of aquatic food sales) and records that are not relevant to the aim of the scoping review.

### 2.2. Scoping Review Characteristics and Assessment

All identified publications were imported into the EndNote software to manage the citations [53]. An Excel file was created with data items, including study characteristics (for instance, author, year of publication, and total Google citation count) [54]. Initially, variations in per capita publications versus per capita aquatic food consumption (kg/year) were determined to understand whether Asian countries with high per capita consumption of aquatic foods are well represented in the scoping review. Further, the number of publications in each year was determined to understand the trend of growth publications in the study period. An evolution of the selected publications stratified by countries and the number of citations received per article (CRPA) in each country were determined to understand which Asian country is most represented in the identified publications and to determine whether doing so attracts the most citations. The CRPA was based on the Google Scholar citation count divided by the number of papers published in each country.

### 2.3. Thematic Clustering

Thematic clustering was performed using the visualisation of similarities (VOS) viewer software version 1.6.18 environment created by Nees Jan van Eck and Ludo Waltman (https://www.vosviewer.com, accessed on 10 September 2022), which is particularly suitable for advancing the intellectual structure of scientific bibliometric mapping to generate research clusters [55,56,57]. Thus, thematic clustering was detected by applying the VOS clustering technique, followed by clustering of the co-occurrence frequencies on text data (title and abstract fields) using association strength [58,59]. Initially, a thesaurus file was created to ensure consistency for different spellings and synonyms in the text data (for instance, social norms would be exchanged with subjective norms). Further, some terms considered irrelevant for analyses were removed, including names of countries and cities. A co-word map was finally produced with a minimum of four occurrences of words in the text data using the VOS mapping technique for displaying clusters [60]. The counting method was set to binary, and the network and overlay visualization scales set to 1.00.

### 2.4. Characterising and Analysing Research Clusters

After associating each publication with a research cluster, descriptive statistics were gathered to show consumers’ age ranges, types of products analysed (e.g., fish, tuna, etc.), and research focus across clusters. Further, the quality of evidence within each research cluster was determined by aggregating individual papers’ scores with the following three criteria: (1) size, (2) quality, and (3) consistency [50]. Table S1 shows the framework for determining the individual scoring of the studies to evaluate the quality of studies in the three-criterion assessment system (validity: 2 questions; rigour: 5; and reliability: 2). For each question, 1 point was given to a “Yes” answer, while “Partially and No” answers scored 0 points.

After the individual papers’ scores were determined, the scores were then aggregated to determine the quality of evidence within each identified cluster. High quality was defined as having scores over 0.75 for all three indicators, i.e., validity, rigour, and reliability; moderate quality was defined as having at least one score below 0.75 but more than 0.5 in at least two out of three indicators; and low quality was defined as having scores less than 0.5 in at least two out of three indicators. It should be noted that reviews and conceptual papers were excluded from the quality assessment. Table S2. briefly shows the criteria for evaluating the size, quality, and consistency of evidence within each cluster.

## 3. Results

### 3.1. Scoping Review Characteristics and Assessment

**Figure 1:**
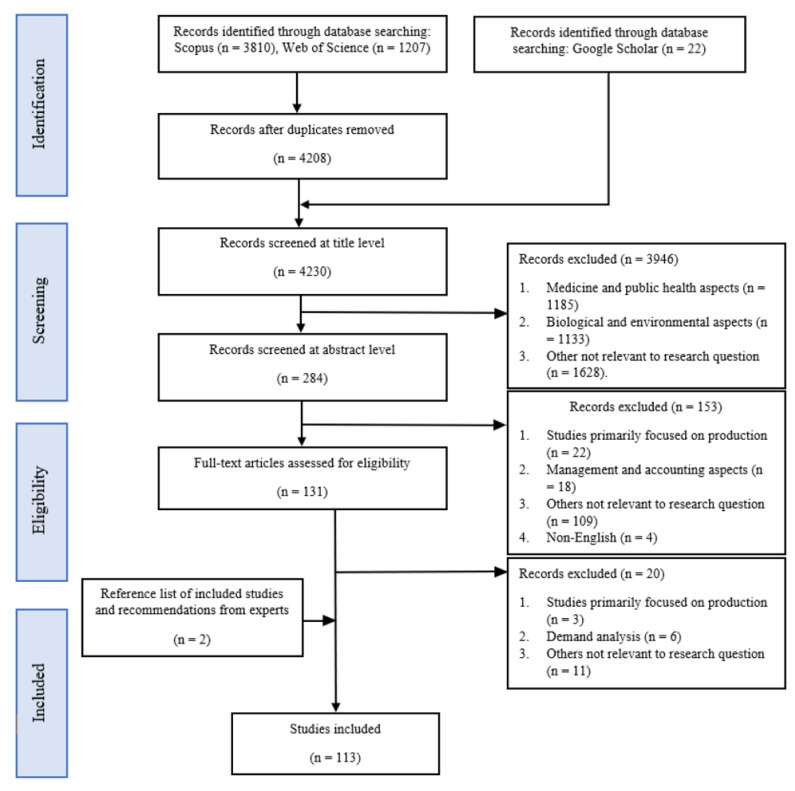
PRISMA–ScR flow diagram indicating the selection process of publications.

**Figure 2:**
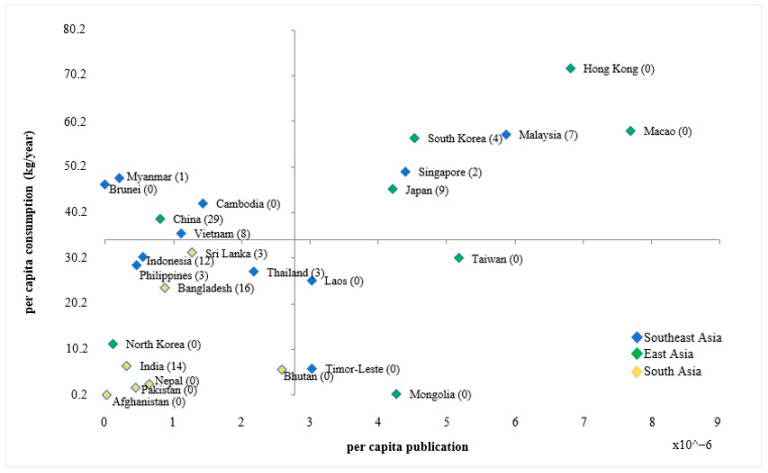
Variations in per capita publication ×10^−6^ versus per capita aquatic food consumption (kg/capita/year). Data on per capita aquatic food consumption was taken from [61]; per capita publication was based on the potential publications to aquatic food consumption behaviour identified through the Scopus database; the bracket indicates the number of publications selected in the scoping review; the Maldives being an outlier was excluded.

**Figure 3:**
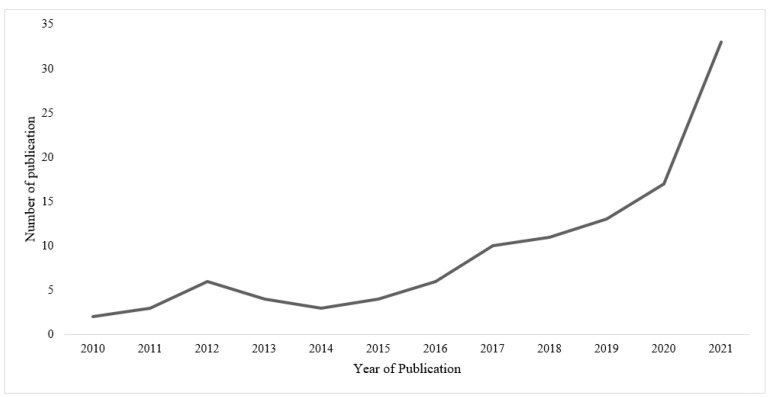
Evolution of the number of publications addressing factors influencing aquatic food consumption behavior among Asian consumers. Publication published after 31st December 2021 was limited and therefore removed in the graph.

After the initial search, 4232 potential records were identified from the three databases. Following the eligibility criteria, the title and abstract of each article were screened and evaluated to either include or exclude the article. At the title level, 3946 articles, and, at the abstract level, 153 articles were excluded that were clearly irrelevant. This process was further repeated by one independent reviewer, and disagreements were resolved through consensus. Full texts of 131 articles were further evaluated to finally include 111 articles. Two additional articles relevant for the scoping review but not identified through this process were recommended by experts. The overall selection process of the 113 identified articles is shown in Figure 1 and is summarized in Table S3, which contains brief information on each publication, including research focus and design. The results from Figure 2 indicate that variation exists in per capita research intensity versus per capita aquatic food consumption (kg/year). Countries such as Hong Kong and Macao have a high per capita aquatic food consumption as well as a high research intensity per capita; however, none were relevant for this scoping review. Further, although China has a comparatively low research intensity per capita, 29 publications were relevant for this study. The results from Figure 3 indicate that 31 publications were recorded in the year 2021 compared to 2 in the year 2010, and publications have more than doubled after 2019.

**Figure 4:**
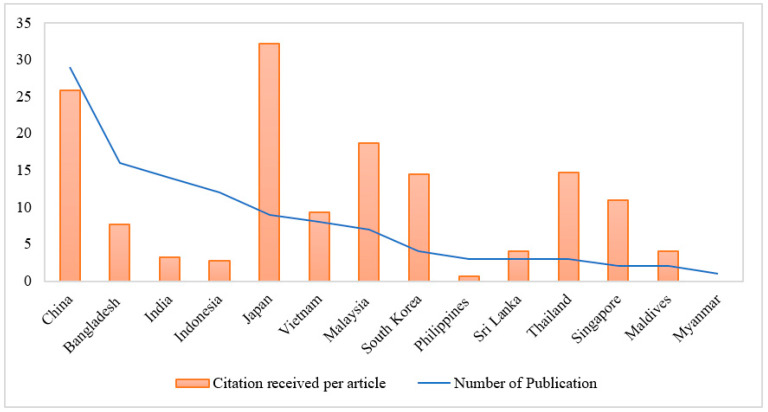
The number of publications and citation impact per publication by research country (2010 to 2022).

Further, the distribution of the identified 113 publications stratified by countries and the number of citations received per article (CRPA) in each country was obtained (Figure 4). The results indicated that Japan, with a lower volume of publications, is responsible for many cited articles.

**Figure 5:**
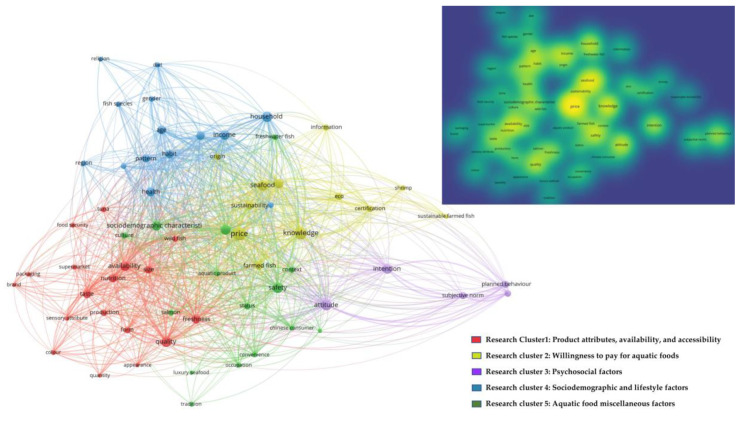
Map of research clusters detected by keyword co-occurrence and density visualisation. Text data (both title and abstract fields) of the identified publication was considered.

Initially, a term map was constructed based on 64 terms that were characterised by five research clusters. Table 1 briefly shows the five identified research clusters, their definitions, and keywords, whereas Figure 5 shows their visualisation and level of saturation. The results from Table 1 and Figure 5 indicate that the term map takes “quality”, “safety”, “price”, “household”, and “attitude” as the core, of which “price” is central. The first cluster roughly corresponds to the product attributes, both intrinsic (i.e., features possessed by aquatic food itself) and extrinsic (packaging, brand) as well as the availability and accessibility of aquatic foods. The second cluster is defined as “willingness to pay for aquatic food products”, as the majority of the publications employed an econometric model to determine the willingness to pay (WTP) for aquatic food products. The third and fourth clusters corresponded roughly to “psychosocial factors, e.g., attitude, subjective norms”, and “sociodemographic and lifestyle factors”, respectively. The fifth cluster we called “aquatic food miscellaneous factors such as food safety, social status” as the keywords overlap with other research clusters; however, the majority of publications with these terms were conducted among Chinese consumers.

### 3.2. Characteristics and Analysis of the Research Clusters

The result of Table 2 indicates that the majority of publications across all research clusters covered consumers aged between 18 and 55 years old. Publications related to “willingness to pay for aquatic foods” analysed various types of aquatic food products; however, the majority of the publications related to the three research clusters, “sociodemographic and lifestyle factors”, “product attributes, availability, and accessibility”, and “psychosocial factors” narrowed to fish in general. All research clusters focused on understanding drivers and barriers to fish consumption, whereas “willingness to pay for aquatic foods” and “product attributes, availability, and accessibility” focused on understanding consumers’ preferences for fish attributes.

Moreover, the quality of the body of evidence ranged from moderate (“product attributes, availability, and accessibility”, “aquatic food miscellaneous factors”, and “psychosocial factors”) to high (“sociodemographic and lifestyle factors”, and “willingness to pay for aquatic foods”). Each research cluster had greater than 10 publications, and thus a large sample size. Remarkably, consistency of research outcome was noted among three research clusters (“product attributes, availability, and accessibility”, “sociodemographic and lifestyle factors”, and “willingness to pay”), whereas it was less consistent for “aquatic food miscellaneous factors” and “psychosocial factors”.

**Research cluster 1: Aquatic food product attributes, availability, and accessibility—23.9% of the identified publications (n = 27).** There was a clear, consistent finding indicating that product attributes, both intrinsic aquatic food product attributes (e.g., taste, flavour, texture, colour, form, and appearance) and external aquatic food product attributes (e.g., quality, packaging, and brand), determine Asian consumers’ aquatic food consumption behaviour. The findings also indicate that taste is dominant over attributes such as health and nutritional value of aquatic foods among the majority of consumers in Asia. Further, availability and accessibility also contribute to aquatic food consumption behaviour [62,63,64,65,66]. For instance, after price, availability and accessibility were ranked the second and third most important factors influencing fish purchasing behaviour among Indian consumers [65].

In general, studies indicated that consumers in Asia preferred wild-caught aquatic foods, especially for their intrinsic attributes [67,68,69,70]. More specifically, the authors of [69] found that consumers in Bangladesh preferred wild-caught fish for freshness and farmed fish for its bigger size. Further, Kitano and Yamamoto [68] indicated that consumers in Japan judge the quality of wild fish as superior to farmed fish and tend to consume wild-caught fish even if the product attributes and conditions between wild and farmed fish were the same.

**Research cluster 2: Willingness to pay for aquatic foods—24.8% of the identified publications (n = 28).** The second research cluster investigated consumers’ preferences and perceptions towards aquatic foods, as well as information and knowledge that influence aquatic food consumption behaviour. The majority of the publications employed an econometric model to determine the willingness to pay (WTP) for aquatic foods (see Table S3). The majority of the studies found that price is a crucial factor influencing aquatic food consumption behaviour among Asian consumers [71,72,73,74]. In general, Asian consumers were willing to pay a higher price for products that have clear package labelling indicating food safety certification [72,75,76,77,78], have been produced (ecolabel) [71,74,79,80,81,82,83,84,85,86], or have country-of-origin [71,82,87] or traceability information [88]. Further, findings suggested that providing additional information and knowledge regarding certification and labelling, as well as processing, could further increase interest and, subsequently, increase willingness to pay for aquatic foods [76,80,81,82,83,85,88,89]. For instance, Yin et al. [88] found that after introducing EU organic certification status in the Chinese shrimp market, consumers’ willingness to pay increased by an average of 84.06%, and further, by providing knowledge introduction, it increased by about 120.16%. Studies have also found that consumers were willing to pay a higher price for ecolabel-farmed aquatic foods when compared with wild-caught aquatic foods [82] and conventionally farmed aquatic foods [80,88].

**Research cluster 3: Psychological factors—16.8% of the identified publications (n = 19).** The majority of the publications in this research cluster (of which 12 out of 19 publications employed structural equation modelling for data analysis) applied social-psychological behavioural theory to explain and predict aquatic food consumption behaviour (see Table S3). According to the theory, the three core components of human behaviour (1. attitude, 2. subjective norms, and 3. perceived behavioural control) together lead to the formation of the intention to consume aquatic foods [90]. In line with behavioural theory, the results from Table 3 show that, although the majority of articles found that attitude, subjective norms, and perceived behavioural control significantly influence the intention to consume aquatic foods, others did not. Further, some articles found attitudes [91,92] as the strongest direct determinants of intention to consume aquatic foods, while others have found subjective norms [93,94,95] and perceived behaviour control [96] to be more important. The underlying cause of having positive or negative attitudes toward aquatic foods tends to be complex, including multiple aquatic food attributes and cues. For instance, Thong and Olsen [92] found that bones and smell have a negative effect on attitude towards fish, while taste, texture, and appearance had important positive impacts on attitudes towards fish. However, product form may affect such attitudes; bones and smell were not perceived as negative factors in terms of attitudes towards dried fish consumption in Bangladesh [97], perhaps because of different food preparation and cooking techniques used for dried compared to fresh fish. Meanwhile, some articles have found that attitudes towards aquatic foods were influenced by perceptions of food safety, environmental concern, convenience and context, and socio-demographic characteristics [98,99,100]; however, having a positive attitude towards aquatic foods might not necessarily translate into eating more fish [101,102]. Finally, notable perceived behavioural control of aquatic food consumption includes affordability, convenience, and accessibility [91,92,96].

**Research cluster 4: Sociodemographic and lifestyle factors—21.2% of the identified publications (n = 24).** This research cluster investigated the sociodemographic and lifestyle factors affecting aquatic food consumption behaviour [108,109]. Studies have indicated that household profile, i.e., type, economy, and size, is one of the important factors affecting aquatic food consumption behaviour [108,109,110,111,112,113]. For instance, household size was found to have a negative correlation to fish consumption [109]. Further, socio-demographic characteristics—age [64,110,114,115,116,117], gender [64,110,116,118], education [110,114,116,118,119,120], marital status [114,115], occupation [119], number of household members [109,115,118], income [108,109,110,115,116,119,121,122], ethnicity [114], religion [116,123], and place of residence [108,114,115,117]—were significantly associated with aquatic food consumption. Overall, older male consumers with higher income and education residing either in an urban setting or coastal region tend to have higher aquatic food consumption levels in Asia. In contrast, other studies have indicated that age [108,118,120], gender [114,119], income [117], marital status [108], number of household members [108], and religion [113] had no significant association with aquatic food consumption.

Lifestyle factors such as health, habit, and dietary pattern also seem to determine aquatic food consumption behaviour. The majority of consumers in Asia preferred eating aquatic foods over alternatives for health benefits [64,109,110,111,113,114,116,124]. Further, Zhou et al. [109] found that the health condition of household members was moderately and positively associated with fish consumption at home. In contrast, Supartini et al. [113] found that the perceived health benefits of fish have no positive impact on seafood consumption behaviour among consumers in Singapore. Further, Zhang et al. [124] found that apart from health motives, dietary habits significantly affected seafood purchasing frequency among consumers from six urban cities in China. However, Huang et al. [106] found that habit strength only influenced the behaviour of consuming fish indirectly via intention, indicating that habits were not formed due to occasional and irregular behaviour, which further indicates that consumers may have inadequate information or experience to make cognitive efforts in fish consumption decision-making. Further, studies have also indicated that purchasing habits, selection of specific fish species, and cooking and eating practices influence aquatic food consumption behaviour [125,126,127,128]. For instance, rural-to-urban migrants in Myanmar fried widely available farmed fish such as tilapia to integrate it into their daily diets instead of cooking small fish species such as anchovies or gourami [*Trichopodus pectoralis*] [128].

**Research cluster 5: Aquatic food miscellaneous factors—13.3% of the identified publications (n = 15).** Closely linked to other research clusters, the majority of the publications in this cluster focused on factors influencing aquatic food consumption behaviour among Chinese consumers. Recurring food safety concerns in Asia have shifted rising middle-class consumers to focus more on the safety of aquatic foods, which is generally reflected in their demand for labelled, wild-caught, imported marine species, particularly from countries including Norway and North America [129,130,131]. Further, diverse consumption habits of aquatic foods were identified, which were explained by numerous factors including place of residence, cultural aspects, social status, and tradition, as well as the importance of convenience, context, and sensory attributes. More specifically, older consumers residing in rural areas preferred domestic and live aquatic foods from local “wet” markets, while younger and wealthier consumers residing in urban areas preferred more imported and convenient aquatic food products from supermarkets or online [129,132,133,134]. Meanwhile, among consumers in Japan, sushi and sashimi are considered convenience and status foods to be eaten in social gatherings [135]. Rising incomes have shifted consumers’ lifestyles and taste preferences among the majority of the middle class residing in urban areas to influence their desires regarding what, how, and where aquatic foods are consumed [131,133,136,137]. For instance, studies have indicated that salmon, lobster, abalone, and sea cucumber are luxury seafood species in China’s high-end market and are often consumed at out-of-home social gatherings. Further, Wang et al. [131] found that Chinese consumers aged 30 years and older and married, with higher-tier incomes, education, and occupations and residing in first-tier cities, have positive beliefs and images of luxury seafood consumption.

## 4. Discussion

This paper aimed to understand the factors influencing aquatic food consumption behaviour among Asian consumers. The VOS clustering of 113 included publications identified five research clusters: 1. product attributes, availability, and accessibility; 2. willingness to pay for aquatic foods; 3. psychosocial factors such as attitude and subjective norms; 4. sociodemographic and lifestyle factors; and 5. aquatic food miscellaneous factors such as food safety and social status. The analysis shows that there are overlapping factors among these five research clusters, suggesting that such behaviour is complex and multifactorial in nature. However, the main factors influencing behaviour are price, knowledge, attitude, quality and safety, and consumers’ household profiles in terms of size, economy, and type. Further, it is notable that publications have more than doubled after the COVID-19 pandemic; however, the majority of them are based on data collected before the COVID-19 pandemic, and only three publications contributed to understanding the impacts of COVID-19 on aquatic food consumption behaviour [73,117,132]. There might be different factors for increasing research interest in understanding Asian consumer preferences and choices towards aquatic foods, including intensification of aquaculture, the dynamic status of the international aquatic food trade, growing urbanisation and globalisation, and the emergence of new innovative technologies for product development. In general, there has been a steep rise in research interest aimed at understanding food choice and behaviour among Asian consumers—a quick search in the Scopus database indicated that the number of publications in the year 2021 was more than doubled when compared to 2015. However, considerable inequalities and anomalies can be seen in the per capita publication rate, the measure of societal investment in the sector versus per capita aquatic food consumption (kg/capita/year), and the measure of the importance of aquatic foods in the diet.

The results from the present study indicate that price is the most important factor influencing aquatic food consumption behaviour among Asian consumers, particularly among poor consumers. Consistently, previous studies have found that, although consumers consider most aquatic food products to be convenient and easy to prepare, price remains the main barrier to eating more aquatic foods [30,138,139]. A previous study found that European consumer food choices were also driven by price rather than sustainability credentials, even when sustainability-related information was understandable and available to consumers [140]. Further, Larson and Story [141] specified that the direct and indirect costs of buying and preparing nutrient-dense foods are important barriers to good nutrition among poorer consumers. In general, foods with higher nutritional value are more expensive in terms of cost per calorie, resulting in wider socioeconomic disparities in diet quality and health [142]. Similarly, aquatic foods are generally expensive yet less filling when compared with other meat alternatives [143]. This might explain why household profiles in terms of type, economy, and size influence aquatic food consumption behaviour among Asian consumers. This finding is in line with previous studies on Western consumers that identified that, as household size increases and income decreases, the probability of consuming aquatic foods declines [144,145]. Therefore, marketing strategies such as nudging and pricing strategies might be particularly effective in influencing aquatic food purchase behaviour in environments where consumption decisions are made [146,147,148]. Further, increasing investment in production research might reduce the direct as well as indirect costs for aquatic foods, making them more competitive with terrestrial animals (e.g., chicken, pork, and beef) to stimulate demand for aquatic foods. These terrestrial substitutes have benefited from centuries of investment compared to aquaculture; however, arguably the ‘yet to be improved’ aquatic species have already demonstrated many intrinsic advantages that R&D investment could further improve [149,150,151,152]. At present, farmed whitefish species, namely tilapia and pangasius, are cheaper than most meat cuts in many Asian countries [153].

Findings from this study suggest that knowledge of, and attitudes toward, aquatic foods are important factors explaining aquatic food consumption behaviour. In general, knowledge about the importance of aquatic foods in a healthy diet creates positive attitudes toward aquatic food. Thus, aquatic food practitioners could focus on promoting the benefits of consuming aquatic foods (e.g., their being healthy, nutritious, tasty, safe, and environmentally sustainable) in comparison to other non-aquatic foods to influence consumption behaviour. Further, quality and food safety tend to have more influence on behaviour when compared with sustainability. These findings are inconsistent with previous studies based on Western consumers that tended to show a higher interest in the sustainability aspects of aquatic foods and a preference for an ecolabel on aquatic foods [26,28,154]. Similarly, a recent study indicated that, unlike Western consumers who exhibit a high demand for ecolabels and sustainable production practices, Chinese consumers emphasize messaging around food safety and quality [31]. Fundamentally, aquatic foods, like any other food item, have the potential to cause disease from viral, bacterial, and parasitic microorganisms under certain conditions, mostly occurring through three sources: 1. processing and preparation; 2. faecal pollution of the aquatic environment; and 3. the natural aquatic environment [155]. However, the microbiological risk from aquatic foods other than raw molluscan shellfish is relatively lower, and such risk mostly results from recontamination or cross-contamination between cooked and raw food products, or contamination during preparation followed by time/temperature mishandling [156]. Additionally, regarding concerns about consuming toxicants such as mercury (Hg) in aquatic foods, recent studies have found that farmed aquatic species such as tilapia, and shrimp generally have the lowest levels of toxicity risk (Hg < 0.15) [157,158]. Therefore, it is recommended to provide credible information regarding the toxicity risks of aquatic foods, invest in educating Asian consumers on safer aquatic food handling practices, and improve food safety knowledge through different channels such as web-based applications [159]. It is also recommended to use credible quality and safety labelling information to create trust and positive attitudes towards aquatic foods [160]. Some argue [161] that integrating information other than the health-related benefits of aquatic foods (e.g., enjoyment and pleasure) and reinforcing positive attitudes towards aquatic foods through arguments that extend beyond health- and nutrition-related benefits might achieve higher levels of compliance with dietary recommendations for aquatic foods. Finally, due to the multifactorial nature of consumption behaviour, it is recommended to consider a multi-faceted approach when developing interventions aimed at increasing aquatic food consumption [162]. For instance, farmed salmon has become increasingly popular in the Chinese diet, latterly through marketing via China’s online sales channels, developing new products targeting individual consumers’ preferences and supported by traceability systems [163,164,165].

This review has some limitations. Firstly, the keywords employed for the literature search might have limited the inclusion of publications, for instance where consumption is not necessarily the focus. The majority of publications employed questionnaires administered either face-to-face or electronically, while few studies employed focus group discussions as exploratory research, and none were longitudinal (see Table S3), thus, limiting casual inference of the findings; in addition, it was not feasible to understand factors influencing behavioural change among Asian consumers. Further, the methodological foundations based on attitudes toward aquatic foods are problematic due to the ubiquitous attitude–behaviour gap [166,167]. Thus, a consumer having a high willingness to pay and positive attitudes toward aquatic foods in surveys might behave quite unpredictively in the marketplace. A recent meta-analysis found that substantial hypothetical bias exists, 21% on average, when measuring willingness to pay for consumer goods [168]. Therefore, methods to determine pricing strategies for aquatic foods should take such biases into account.

Further, only publications in English were reviewed, thus limiting our understanding. We employed VOS viewer software for visualising bibliometric networks; however, integrating VOS viewer with other tools such as SciMAT and Citespace might have increased the advantages and offered new opportunities for better interpretation of the scientific map. Further, the qualitative descriptive process involved in the interpretation of such a map is complex and such interpretation is prone to unintentional bias. Thirdly, the inclusion of two cross-national comparative studies that compared aquatic food consumption behaviour among consumers in Asia and Western countries might have influenced the findings.

Finally, consumer sensory tests were under-represented in this review, and only one study attempted to integrate consumer sensory taste testing; however, the study was subjected to poor methodological quality (see Table S3). Thus, the findings of research cluster 1: product attributes, availability, and accessibility—intrinsic aquatic food product attributes influencing behaviour, were based on a proxy of sensory perception, i.e., ‘to what extent does consumer agree or disagree that the aquatic foods have a great taste’ that might have methodological limitations [21]. However, such attitudinal statements tend to evaluate aquatic foods with some degree of satisfaction or dissatisfaction with either liking or disliking aquatic foods [169].

## 5. Conclusions and Future Research

In conclusion, the results from this study identified five clusters of publications— “product attributes, availability, and accessibility”, “willingness to pay for aquatic foods”, “psychosocial factors”, “sociodemographic and lifestyle factors”, and “miscellaneous factors”—that provided insights into multiple interacting factors influencing aquatic food consumption behaviours factors among Asian consumers. The main factor influencing aquatic food consumption behaviour among Asian consumers is price, as shown by the large majority of Asian consumers. Further, knowledge of, and attitudes toward, perceived quality and safety of aquatic foods were identified as important but mediated by household characteristics. Sustainable production practices, country of origin, and ecolabels were found to be less influential on consumption behaviour.

There are a growing number of research interests in understanding aquatic food consumption behaviour among Asian consumers; however, identified publications present a similar pattern involving a narrow tunnel-view of their respective disciplines toward the comprehension and prediction of complex aquatic food consumption behaviours in a real-world setting. At present, disciplines including sociology, marketing, food science, and economics have at least partially answered the central questions in aquatic food consumption behaviour research, i.e., why, when, and where do consumers in Asia consume aquatic foods? For instance, most of the available economic research investigated the price, aquatic food attributes, and household expenditure aspects of aquatic food consumption behaviour among consumers in Asia. Thus, future studies should consider more multidisciplinary research based on a deductionist approach [21] to better understand and predict multiple factors and their interactions that influence aquatic food consumption behaviour among Asian consumers. Arguably, there is an urgent need for more studies that address the gap between more intrinsic product characteristic perceptions, such as sensory characteristics, and more extrinsic aquatic food product attributes, such as packaging and labelling. Further, such an attempt should cross-fertilize with economics and psychology to make real progress in understanding complex aquatic food consumption behaviour in its totality.

Secondly, some studies have indicated that aquatic food consumption behaviour might be a habitual behaviour that is performed repeatedly and routinely without conscious evaluation of possible outcomes. Thus, the high consumption of aquatic foods among consumers in some parts of Asia might simply be due to a strong habit of eating aquatic foods formed by aggregated satisfactory past experiences through cultural predisposition that eventually become habitual. However, studies with in-depth analysis of habits influencing aquatic food consumption are under-represented, and in only one study was it possible to observe how individuals’ everyday aquatic food consumption practices changed when they shifted from rural to urban areas (see Table S3).

Generally, Asian consumers seem to have positive attitudes towards aquatic foods; however, efforts to bridge the gap between attitudes and behaviour towards aquatic food consumption could be developed by employing food safety, price, quality, convenience, and knowledge of aquatic foods as other relevant factors to behavioural decision-making criteria. Further, attitudinal ambivalence towards aquatic foods seems to have emerged, especially among consumers from urban areas, to influence the relationship between attitudes and behaviour towards aquatic food consumption. For instance, most urban consumers in Asia seem to prefer live and whole fish that is wild caught; on the other hand, they also prefer aquatic foods that are sustainable, convenient, and easily available at a competitive price. The focus on the effect of ambivalence on aquatic food consumption behaviour among consumers residing in urban settings is lacking, and this aspect could have important practical implications for practitioners who are involved in the promotion of change in aquatic food consumption behaviour. Finally, future studies might also consider conducting a systematic review with meta-analysis to understand how various but specific factors identified by this study influence aquatic food consumption behaviour among Asian consumers.

## Figures and Tables

**Table 1:** Defining research clusters based on keywords used in aquatic food consumption literature.

Cluster-ID/Colour	Research Clusters	Definition of Cluster	Keywords (Links, Total Link Strength, Occurrences)
**C1/Red**	Product attributes, availability, and accessibility	Product attributes both, intrinsic and extrinsic, as well as availability and accessibility of aquatic food products.	Quality (56, 207, 25), availability (53, 160, 20), freshness (57, 147, 18), taste (53, 136, 16), nutrition (49, 121, 15), size (50, 129, 14), form (47, 93, 11), production (36, 68, 10), wild fish (42, 79, 8), tuna (39, 63, 8), colour (25, 48, 7), food security (36, 52, 6), supermarket (36, 54, 6), sensory attributes (38, 55, 5), packaging (31, 41, 5), appearance (28, 43, 4), quantity (28, 39, 4), brand (21, 30, 4)
**C2/Yellow**	Willingness to pay for aquatic foods	The majority of the publications are related to Asian consumers’ willingness to pay (WTP) for aquatic foods.	Price (60, 281, 41), knowledge (57, 220, 34), seafood (55, 168, 24), fish product (51, 152, 20), farmed fish (50, 107, 15), sustainability (48, 106, 14), eco (34, 72, 12), certification (42, 90, 11), origin (35, 61, 9), information (29, 52, 9), shrimp (30, 44, 7), sustainable farmed fish (22, 34, 5)
**C3/Purple**	Psychosocial factors	Psychosocial factors influencing aquatic food consumption behaviour.	Attitude (57, 202, 28), intention (52, 134, 22), subjective norms (34, 79, 12), planned behaviour (25, 68, 12), behavioural control (28, 63, 10)
**C4/Blue**	Sociodemographic and lifestyle factors	Sociodemographic and lifestyle factors influencing consumption behaviour towards aquatic foods.	Household (50, 165, 24), income (50, 167, 20), health (50, 143, 18), habit (52, 141, 17), age (43, 135, 17), education (51, 142, 16), pattern (45, 122, 15), economy (35, 60, 8), urban area (37, 63, 8), gender (34, 60, 8), fish species (33, 51, 8), region (33, 49, 8), diet (30, 52, 7), religion (21, 30, 5)
**C5/Green**	Aquatic food miscellaneous factors	Closely linked to other research clusters, the majority of the publications are related to Chinese aquatic consumption.	Safety (50, 183, 24), city (59, 173, 23), sociodemographic characteristics (55, 136, 20), status (43, 87, 11), culture (35, 54, 9), context (34, 47, 9), freshwater fish (38, 66, 8), salmon (32, 45, 8), convenience (41, 69, 7), aquatic product (36, 58, 7), occupation (35, 60, 7), Chinese consumer (35, 50, 6), tradition (20, 24, 5), shellfish (32, 42, 4), luxury seafood (20, 24, 4)

**Table 2:** Characteristics of the five research clusters and their quality assessment.

Variables		Product Attributes, Availability, and Accessibility	Willingness to Pay for Aquatic Food	Psychosocial Factors	Sociodemographic and Lifestyle Factors	Aquatic Food Miscellaneous Factors
Consumers’ age range, median (IQR)		37(21.8)	43.2(13.4)	30(28.25)	43.5(23.7)	47(25.7)
Types of products analysed %(*n*)	Fish in general	34.6(9)	7.4(2)	47.4(9)	62.5(15)	14.3(2)
	Seafood in general	7.7(2)	26(7)	10.5(2)	12.5(3)	35.7(5)
	Aquaculture products	11.6(3)	22.2(6)		4.2(1)	
	Processed fish products	19.2(5)	3.7(1)	15.8(3)	8.3(2)	21.4(3)
	Tuna	15.4(4)	3.7(1)		4.2(1)	
	Salmon		7.4(2)			21.4(3)
	Shrimp	3.8(1)	11.1(3)	5.2(1)		
	Shellfish	7.7(2)	3.7(1)	5.2(1)		7.1(1)
	Labelling		11.1(3)	10.5(2)		
	Others		3.7(1)	5.3(1)	8.3(2)	
Research focus %(*n*)	Drivers and barriers to fish consumption	61.5(16)	48.2(13)	89.4(17)	75(18)	71.4(10)
	Consumer preferences for fish attributes	30.8(8)	51.8(14)	5.3(1)	12.5(3)	7.1(1)
	Drivers and barriers to seafood consumption	3.8(1)				14.3(2)
	Drivers and barriers to shellfish consumption	3.8(1)			8.3(2)	7.1(1)
	Drivers and barriers to seaweed consumption			5.3(1)		
	Fish consumption practices				4.2(1)	
Validity		0.92	0.94	0.72	0.77	0.89
Rigour		0.88	0.95	0.88	0.90	0.94
Reliability		0.67	0.76	0.68	0.70	0.64
Quality ^a^		Moderate	High	Moderate	High	Moderate
Size		Large	Large	Large	Large	Large
Consistency		Consistent	Consistent	Inconsistent	Inconsistent	Consistent

Note: **^a^** Quality was based on the scores from validity, rigour, and reliability with scores >0.75 in all indicators considered high and at least one score <0.75 considered moderate [50]; three review or conceptual papers were not given a quality score; IQR: interquartile range.

**Table 3:** Attitude, subjective norms, and perceived behavioural control influence the intention to consume aquatic foods.

Association between Psychosocial Factors	Significant Influence	No Significant Influence
Attitude→Intention	[91,92,93,94,97,100,103,104]	[105,106]
Subjective norms→Intention	[92,93,94,95,97,105]	[91,104,106]
Perceived behavioural control→Intention	[91,94,95,96,106,107]	[92,93,97,104,105]

## Data Availability

No new data were created or analyzed in this study. Data sharing is not applicable to this article.

## Supplementary Materials

The following supporting information can be downloaded at https://www.mdpi.com/article/10.3390/foods11244043/s1: Table S1: Framework for determining identified publication’s individual score; Table S2: Criteria for evaluating the size, quality, and consistency within the identified research clusters. Table S3: Information on selected publications.
