# Supplementary material for: Factors Influencing Consumption Behaviour towards Aquatic Food among Asian Consumers: A Systematic Scoping Review
Source: Foods. 2022 Dec 14;11(24):4043. doi: 10.3390/foods11244043 (PMC9777948; doi:10.3390/foods11244043)
Supplement: Supplementary file 1 [file foods-11-04043-s001.zip › Supplementary Materials (2).pdf]

Supplementary Material

Table S1. Framework for determining identified publication's individual score (adopted from the Ref. [1]**).**

Indicator	Criteria
Validity	1. 'Are the findings substantiated by the data and has consideration been given to limitations of the methods that may have affected the results?'<br>2. 'Are there issues in applying the method to some research question(s), i.e., was the methodology adequate for the research question?'
Rigour	1. Is the context or setting adequately described?<br>2. Is (are) the research question(s) clear?<br>3. Is the method used appropriately to answer the research question(s)?<br>4. Is the method applied correctly?<br>5. Is there evidence that the data collection was rigorously conducted to ensure confidence in the findings?
Reliability	1. Is the data analysis rigorously conducted to ensure confidence in the findings?<br>2. Is the methodology adequately described to ensure confidence in the findings?

Table S2. Criteria for evaluating the size, quality, and consistency within the identified research clusters (adapted from the Ref. [1]**).**

Indicators	Categories	Criteria
Size	Large	>10 documents
	Medium	5≥ and 10≤
	Small	<5
Quality	High	>0.75 scores across all three indicators i.e., validity, rigour, and reliability
	Moderate	<0.75 at least one score, but >0.5 at least two scores
	Low	<0.5 scores at least two scores
Consistency	Consistent	A range of studies with identical, or similar conclusions.
	Inconsistent	Different studies point to a range of conclusions. For instance, different designs or methods applied in different contexts resulting contrasting findings.

Table S3. Information on selected publications.

[Research Cluster 1 (RED) = **Product attributes, availability, and accessibility;** Research Cluster 2 (Yellow) = **Willingness to pay for aquatic foods;** Research Cluster 3 (Purple) = **Psychosocial factors;** Research Cluster 4 (Blue) = **Sociodemographic and lifestyle factors;** Research Cluster 5 (Green) = **Aquatic food miscellaneous factors**]

Ref.	Author(s)	Year	Focus	Country	Research design, sampling, data collection method and period	Sample size	Sample composition	Age range	Data analysis method	Type of products analysed
[2]	Chuenba and Sawmong	2018	Determinants of canned seafood product	Thailand	Cross-sectional, systematic random, face-to-face interview, Sep 2016	400	Consumers who purchased and consumed canned and processed seafood	21-60 and older	Structural equation modelling	Canned and processed seafood
[3]	Devadawson et al.	2015	Drivers and barriers to fish consumption	Sri Lanka	Cross-sectional, purposive, face-to-face interview, Dec 2013 – Oct 2014	150	Consumer who purchases and consume fish	n/a	Quantitative descriptive analysis	Fish and seafood in general
[4]	Das et al.	2013	Drivers and barriers to fish consumption	India	Cross-sectional, multistage stratified random, face to face interview, June-July 2011	160	Fish consumers	25 and younger – 45 and older	Rank-based quotient	Fish and seafood in general
[5]	Akter et al.	2019	Drivers and barriers to fish consumption	Bangladesh	Cross-sectional, purposive, face-to-face interview, Feb 2018	100	Households participated in USAID funded projects	n/a	One wat ANOVA and Tukey's post hoc test	Fish in general
[6]	Chokenu et al.	2019	Drivers and barriers to fish consumption	Thailand	Cross-sectional, multi-stage simple random, face-to-face interview, Oct 2015-Dec 2015	700	Consumer purchased processed fish products	n/a	Structural equation modelling	Processed fish products
[7]	Chuenba et al.	2021	Drivers and barriers to fish consumption	Thailand	Cross-sectional, systematic random, face-to-face interview, n/a	400	All types of consumers	n/a	Structural equation modelling	Canned tuna
[8]	Hossain et al.	2021	Consumers' preferences for fish attributes	Bangladesh	Cross-sectional, stratified simple random, self-administered, Nov 2017-Feb 2018	300	All types of consumers	n/a	Hedonic price model	Tilapia
[9]	Hossain	2021	Consumers' preferences for fish attributes	Bangladesh	Cross-sectional, simple random, face-to-face interview, Dec 2020	200	All types of consumers	35 and younger-45 and older	Hedonic price model	Fish in general
[10]	Intyas and Primyasta	2020	Consumers' preferences for fish attributes	Indonesia	Cross-sectional, convenience, face-to-face interview, n/a	38	Consumers of preserved Mackerel tuna fish products	20 and younger-49	Cross-tabulation, multiple	Mackerel tuna fish

									regression, F test and t-test	
[11]	Mitra et al.	2021	Consumers' preferences for fish attributes	Bangladesh	Cross-sectional, simple random, face-to-face interview, Feb 2019 – Mar 2020	138	Consumers who buy capture and culture fishes	n/a	Logit model, hedonic pricing model	Capture and culture fish
[12]	Tan et al.	2017	Consumers' preferences for fish attributes	Philippines	Cross-sectional, multistage stratified random, face to face, NA	378	All types of consumers	20 and younger-60 and older	Descriptive statistics, ordinal least square regression	Milkfish
[13]	Uddin et al.	2019	Drivers and barriers to fish consumption	Bangladesh	Cross-sectional, purposive random, face to face, NA	150	All types of consumers	15 and younger-55 and older	Descriptive statistics, Logit model	Pangas and Tilapia Fish
[14]	Wang et al.	2021	Consumers' preferences for fish attributes	China	Cross-sectional, convenience, web-based questionnaire, Dec 2016	981	All types of consumers	32.1±n/a	Descriptive statistic, binary logistic regression	Lobster
[15]	Wang and Somogyi	2018	Drivers and barriers to shellfish consumption	China	Cross-sectional, quota, web-based questionnaire, Dec 2016	643	All types of consumers	18 – 40 and older	Descriptive statistics, structural equation modelling	Shellfish in general
[16]	Ahmed et al.	2011	Drivers and barriers to fish consumption	Malaysia	Cross-sectional, simple random, face-to-face interview, Dec 2007	700	Household member responsible for purchasing food	18 and younger – 55 and older	Descriptive statistics, Logit model	Fresh fish
[17]	Omar et al.	2011	Drivers and barriers to fish consumption	Malaysia	Cross-sectional, convenience, self-administered, n/a	212	Fish snack consumers	n/a	Cross-tabulation, correlation, t-test	Fish snack
[18]	Sajeev et al.	2021	Drivers and barriers to fish consumption	India	Cross-sectional, purposive random, face-to-face interview, 2019-2020	97	Consumers purchasing fish online	39.28±11.13	Cross-tabulation, ordinal alternative least square	Fish in general
[19]	Wijayanto et al.	2021	Drivers and barriers to fish consumption	Indonesia	Cross-sectional, cluster, self-administered, 2019	720	All types of consumers	32 and younger-59 and older	Regression and correlational analysis	Fresh shrimp
[20]	Linh et al.	2019	Drivers and barriers to fish consumption	Vietnam	Cross-sectional, convenience, self-administered, n/a	300	All types of consumers	20-35 and older	Word association, factor analysis,	Fish sauce products
[21]	Castro et al.	2016	Consumers' preferences for fish attributes	Philippines	Two stages, focus group, convenience, Jun-Jul 2015 + cross-sectional, convenience, face-to-face interview, n/a	69 (focus group) + 204 (survey)	Consumers who purchase canned tuna	16-75	Content analysis + hierarchical and k-mean cluster analysis, probit model	Canned tuna

[22]	Izzhati et al.	2018	Drivers and barriers to fish consumption	Indonesia	Two stages, Focus group + conjoint analysis, Cross sectional, convenience, self-administered, n/a	n/a + 100	All types of consumers	18-60	Content analysis + cross tabulation, corelation	Smoked fish
[23]	Rejula et al.	2021	Drivers and barriers to fish consumption	India	Cross-sectional, purposive random, face-to-face interview, Dec 2018-Mar 2020	400	Fish consuming households	19-77	Cross-tabulation	Fish in general
[24]	Mugaonkar et al.	2011	Consumers' preferences for fish attributes	India	Two-stage, convenience, face to face interview + sensory evaluation, Jan-Feb 2009	140 (survey) + 50 (sensory test)	All types of consumers + willingness to taste value-based products	25 and younger – 35 and older	Cross-tabulation, factor analysis	Fish in general
[25]	Zaeema and Hassan	2016	Drivers and barriers to fish consumption	Maldives	Cross-sectional, convenience, self-administered, n/a	450	All types of consumers	18 to 50 and older	Structural equation modelling, one way ANOVA	Canned tuna
[26]	Sajiki and Lu	2022	Drivers and barriers to fish consumption	Japan	Cross-sectional, convenience, web-based questionnaire, Nov 2018	1000	Household member responsible for purchasing seafood	20 or older	Conditional logit model and random parameter logit model	Raw fish
[27]	Kitano and Yamamoto	2020	Drivers and barriers to fish consumption	Japan	Cross-sectional, convenience, web-based questionnaire, n/a	493	Consumers responsible for household's fresh food purchasing decision	50.89±16.0	Factor analysis, Poisson model, ordinal least square	Fish in general
[28]	Mugaonkar et al.	2013	Drivers and barriers to fish consumption	India	Cross-sectional, simple random, face-to-face interview, Jul-Oct 2012	120	Household member responsible for food purchasing/cooking	n/a	Ordered probit model	Fish and seafood in general
[29]	Alam and Alfnes	2020	Consumers' preferences for fish attributes	Bangladesh	Cross-sectional, convenience, face-to-face interview, Feb-Mar 2016	400	Household heads responsible for at least 50% of food purchasing	25-60	Mixed logit model	Pangasius and Rohu
[30]	Ariji	2010	Consumers' preferences for fish attributes	Japan	Cross-sectional, convenience, web-based questionnaire, Mar 2009	12000	All types of consumers	20-40 and older	Conjoint analysis	Bluefin tuna
[31]	Hoque	2021	Consumers' preferences for fish attributes	Bangladesh	Cross-sectional, convenience, face-to-face interview, Aug -Oct 2018	490	Household member responsible for buying fish	20-69	Multinominal logit model, latent class logit model	Fish in general
[32]	Mohammed et al.	2021	Consumers' preferences for fish attributes	Bangladesh	Cross-sectional, stratified random, face-to-face interview, Aug-Oct 2019	660	Household member responsible for buying fish	33.93±8.97	Descriptive analysis, Rank-ordered logit model	Shrimp
[33]	Hoque and Alam	2020	Drivers and barriers to fish consumption	Bangladesh	Cross-sectional, stratified random, face-to-face, Mar-Apr 2018	498	households who prefer fish and are responsible for buying for household	20-70 and older	Structural equation modelling	Farmed fish in general

[34]	Hoque et al.	2021	Drivers and barriers to fish consumption	Bangladesh	Cross-sectional, random, face-to-face interview, Jan-Apr 2019	1053	All types of consumers	41.25±13.28	Structural equation modelling	Farmed fish in general
[35]	Hori et al.	2020	Consumers' preferences for fish attributes	Japan	Cross-sectional, convenience, web-based questionnaire, 2017 and 2019	3000	All types of consumers	20 and older	Factor analysis, multiple regression analysis, marginal willingness to pay	Fish and seafood in general
[36]	Karnad et al.	2021	Drivers and barriers to fish consumption	India	Two stage, semi-structured interview + cross-sectional, convenience, Jun-Oct 2018	40 (interview)+ 531 (survey)	Seafood eaters	20 and younger-60 and older	Content analysis + crosstabulation	Fish and seafood in general
[37]	Kim and Lee	2018	Drivers and barriers to fish consumption	South Korea	Cross-sectional, simple random, web-based questionnaire, Jun-Jul 2017	2773	Household member responsible for food purchasing/cooking	20-69	Descriptive statistics, ordered probit model	Seafood in general
[38]	Murakami et al.	2017	Drivers and barriers to fish consumption	Japan	Cross-sectional, simple random, web-based questionnaire, Dec 2015	1148	All types of consumers	20-69	Post-hoc test, logistic regression, t-test	Fish and shellfish in general
[39]	Prince and Wahid	2020	Drivers and barriers to fish consumption	Bangladesh	Cross-sectional, stratified random, face to face interview, Mar-Jun 2020	320	Organic fish consumers	24-60 and older	Structural Equation modelling	Organic fish
[40]	Zheng et al.	2021	Consumers' preferences for fish attributes	China	Cross-sectional, simple random, face-to-face interview, 2015	1017	All types of consumers	37.20±13.51	Descriptive statistics, random parametric logit model, ordinal least square regression model	Salmon
[41]	Omar et al.	2021	Drivers and barriers to fish consumption	Malaysia	Cross sectional survey, purposive, self-administered, NA	307	Sardine consumers	NA	Structural equation modelling	Canned sardine
[42]	Sayeed et al.	2021	Consumers' preferences for fish attributes	Singapore	Cross-sectional, stratified, web-based questionnaire, Mar-Apr 2021	312	All types of consumers	18-55 and above	Descriptive statistics, conditional logit model	Mud crabs
[43]	Tan	2017	Drivers and barrier to fish consumption	Philippines	Cross-sectional, multistage stratified random, face-to face interview, n/a	378	All types of consumers	20 and younger-60 and older	Cross-tabulation, One-way ANOVA	Milkfish
[44]	Uchida et al.	2014	Consumers' preferences for fish attributes	Japan	Cross sectional, convenience, web-based questionnaire, Mar 2009	3370	All types of consumers	42.5±10.6	Mixed logit model	Eco labelled seafood

[45]	Wakamatsu et al.	2017	Consumers' preferences for fish attributes	Japan	Cross-sectional, convenience, face-to-face interview, Jun 2009	159	Females who are responsible for purchasing/cooking food	40 and younger-50 and older	Latent class, multinominal logit, Chi-Square	Eco labelled seafood
[46]	Wakamatsu and Miyata	2017	Consumers' preferences for fish attributes	Japan	Cross-sectional, convenience, web-based questionnaire, Nov 2015	2378	General domestic seafood consumers	15-60	Random utility model, conditional logit model	Seafood in general
[47]	Xu et al.	2012	Consumers' preferences for fish attributes	China	Cross-sectional, convenience, face-to-face interview, Nov 2009	386	All types of consumers	39.53±13.38	Descriptive analysis, probit regression	Seafood in general
[48]	Xuan et al.	2021	Drivers and barriers to fish consumption	Vietnam	Cross-sectional, stratified random quota, face to face interview, Mar-Apr 2019	754	All types of consumers	36.79±12.89	Cross-tabulation, ordered logistic regression, multiple indicators multiple causes model	Shrimp
[49]	Xuan	2021	Consumers' preferences for fish attributes	Vietnam	Cross-sectional, random quota, face-to-face interview, Mar-Apr 2019	353	Household member responsible for purchasing/cooking food	37.38±11.43	Multinominal logit model, mixed-logit model	Aquaculture products
[50]	Yi	2019	Drivers and barriers to fish consumption	South Korea	Cross-sectional, stratified, web-based questionnaire, Nov-Dec 2018	960	All types of consumers	20-60 and older	Descriptive statistics, structural equation model	Aquaculture products
[51]	Yi	2019	Drivers and barriers to fish consumption	South Korea	Cross-sectional, convenience, web-based questionnaire, n/a	1000	All types of consumers	20-60 and older	Factor analysis, double bounded dichotomous choice model, contingent valuation model	Red seabream
[52]	Yin et al.	2020	Consumers' preferences for fish attributes	China	Two stages, focus group: convenience, Oct-Nov 2016 + cross-sectional, simple random, face-to-face interview, Jan - Mar 2018	Focus group n/a + survey 996	Household member responsible for purchasing and had purchased seafood within the last month	18 and older	n/a + descriptive statistics, generalised mixed logit model	White shrimp
[53]	Zhang et al.	2020	Consumers' preferences for fish attributes	China	Cross-sectional, purposive, web-based questionnaire, May -Jun 2018	372	Seafood and salmon consumers	18 and older	Mixed logit model, conditional logit model, equality constrained latent class	Salmon

[54]	Peng et al.	2021	Drivers and barriers to fish consumption	China	Secondary data analysis, face-to-face interview, 2004, 2006, 2009	13,386	Households from the China Health and Nutrition Survey (CHNS)	48.96±15.22	Chi-Square, Tobit model	Seaweed
[55]	Ghosh et al.	2018	Drivers and barriers to fish consumption	India	Cross-sectional, simple random, face to face interview, Dec 2014 - Feb 2015	120	All types of consumers	15 – 60 and older	Rank-based quotient	Ready to eat fish products
[56]	Abidin et al.	2021	Drivers and barriers to fish consumption	Indonesia	Cross-sectional, convenience, web-based questionnaire, n/a	144	Fish consumers	20-23	Structural equation modelling	Fishery functional foods
[57]	Chen and Wang	2021	Drivers and barriers to fish consumption	China	Cross-sectional, random stratified, web-based questionnaire, Jun 2020	1096	All types of consumers	30 and younger – 60 and older	Structural equation modelling	Wild freshwater fish
[58]	Dai et al.	2021	Drivers and barriers to fish consumption	China	Cross-sectional, convenience, web-based questionnaire, May – July 2019	4221	All types of consumers	18 and older	Multinomial ordinal logistic regression model	Aquatic products
[59]	Fiandari et al.	2019	Drivers and barriers to fish consumption	Indonesia	Cross-sectional, Convenience, face-to-face interview, Jun 2017-Feb 2018	365	Consumed fish for at least one year	17 and older	Structural equation modelling	Fish in general
[60]	Gajaria and Mantri	2021	Drivers and barriers to seaweed consumption	India	Cross-sectional, convenience, web-based questionnaire, Jun 2020 – Jun 2020	310	All types of consumers	15-50 and older	Student's t-test, ANOVA	Seaweed
[61]	Ghifarini et al.	2018	Drivers and barriers to fish consumption	Indonesia	Cross-sectional, Purposive, web-based questionnaire, Jan- Feb 2016	120	All types of consumers	30 and younger-40 and older	Structural equation modelling	Shrimp
[62]	Li and Zhong	2017	Drivers and barriers to fish consumption	China	Cross-sectional, convenience, web-based questionnaire, May-Jun 2016	337	All types of consumers	18 and younger-60	Structural equation modelling	Green aquatic products
[63]	Novita and Rowena	2019	Drivers and barriers to fish consumption	Indonesia	Cross-sectional, convenience, self-administered, n/a	307	All types of consumers	20-50	Structural equation modelling	Fish in general
[64]	Pethiyagoda and Olsen	2013	Drivers and barriers to fish consumption	Sir Lanka	Cross sectional, convenience, face to face interview, NA	207	All types of consumers	33±12	Confirmatory factor analysis and structural equation modelling	Fish in general
[65]	Quan et al.	2014	Drivers and barriers to fish consumption	China	Cross sectional, simple random, face to face interview, Nov 2009	386	All types of consumers	21-60	Structural Equation modelling	Eco labelled fish
[66]	Siddique	2012	Consumers' preferences for fish attributes	Bangladesh	Cross-sectional, convenience, face to face, Feb-Apr 2011	558	All types of consumers	18-45	Exploratory factor analysis, multiple regression	Dry fish
[67]	Thong and Olsen	2012	Drivers and barriers to fish consumption	Vietnam	Cross sectional, convenience, face- to	208	All types of consumers	25-55	Descriptive statistics, structural	Fish in general

					face interview, Aug 2006				equation modelling	
[68]	Wang and Somogyi	2019	Drivers and barriers to fish consumption	China	Cross-sectional, quota stratified random, web-based questionnaire, Dec 2016	643	All types of consumers	18-40 and older	Descriptive statistics, structural equation modelling	Shellfish in general
[69]	Fauziyah	2020	Drivers and barriers to fish consumption	India	Cross-sectional, convenience, face-to face interview, Apr 2019	150	Adolescents	15-17	Chi-Square, multiple logistic regression	Fish in general
[70]	Junaidi et al.	2020	Drivers and barriers to fish consumption	Indonesia	Cross-sectional, convenience, web-based questionnaire, n/a	55	All types of consumers	26-40	Structural equation modelling	Fresh fish
[71]	Ho Huy et al.	2013	Drivers and barriers to fish consumption	Vietnam	Cross-sectional, convenience, face-to-face interview, n/a	466	Eat fish at least once a week	18-76	Structural equation modelling	Fish and seafood in general
[72]	Duy and Ai	2019	Drivers and barriers to fish consumption	Vietnam	Cross-sectional, systematic random, self-administration, n/a	299	All types of consumers	n/a	Factor analysis, linear regression model	Fish and seafood in general
[73]	Rotuauli et al.	2020	Drivers and barriers to fish consumption	Indonesia	Cross-sectional, accidental, self-administered, n/a	100	All types of consumers	21-69	Cross-tabulation, corelation	Freshwater fish
[74]	Muganokar et al.	2017	Drivers and barriers to fish consumption	India	Cross-sectional, convenience, face to face interview, 2012	120	Fish consumers	15 and younger – 45 and older	Rank-based Quotient	Pangasius
[75]	Danso et al.	2017	Consumers' preferences for fish attributes	Vietnam	Cross-sectional, simple random, face-to-face interview, 2014	136	Household member responsible for food purchasing/cooking	25 and younger-65 and older	Latent class model, conditional logit and random parameters logit models.	Fish products
[76]	Ahmad et al.	2016	Drivers and barriers to fish consumption	Malaysia	Cross-sectional, two-stage proportionate stratified, face-to-face interview, Feb 2008 – May 2009	2675	Household member responsible for food purchasing/cooking	43.4±16.2	Student's t-test, one way ANOVA, Mann-Whitney U and Kruskal-Wallis H test	Fish in general
[77]	Arthatiani et al.	2021	Socio-economic determinants of preserved fish consumption	Indonesia	Data were taken from the National socio-economic survey for Indonesia, 2019	96,360	All types of consumers	40 and younger – 55 and older	Probit regression model	Preserved fish
[78]	Devadawson et al.	2015	Drivers and barriers to fish consumption	Sri Lanka	Cross-sectional, stratified random, face-to-face interview, 2014	1777	Fish consumers	25-75	Cross-tabulation, Kruskal – Wallis test	Fish in general

[79]	Fu et al.	2019	Drivers and barriers to fish consumption	China	Cross-sectional, simple random, face-to-face interview, Jun 2017-Jan 2018	451	All types of consumers	36.36±17.74	Mann-Whitney U-tests, Kruskal-Wallis tests	Asian horseshoe crabs
[80]	Haque et al.	2019	Drivers and barriers to fish consumption	Bangladesh	Cross-sectional, convenience, quota-based, self-administered, 2018	120	Household member responsible for purchasing seafood	35 and younger-60 and older	Descriptive statistics and chi-square	Fish in general
[81]	Hoa and Kare	2017	Drivers and barriers to fish consumption	Vietnam	Cross-sectional, purposive, Face-to-face interview, 2010	413	Teenagers	12-20	Factor analysis, t-test and ANOVA	Fish in general
[82]	Islam et al.	2018	Drivers and barriers to fish consumption	Bangladesh	Cross-sectional, convenience, self-administered, n/a	658	Consumers responsible for their household purchasing	16 to 65 and older	Factor analysis, cross-tabulation, F-test	Fish in general
[83]	Kashem et al.	2021	Drivers and barriers to fish consumption	Bangladesh	Cross-sectional, convenience, web-based questionnaire, Jul-Aug 2020	300	All types of consumers	24 and younger – 45 and older	Descriptive statistic, correlation	Fish in general
[84]	Lee and Nam	2019	Drivers and barriers to fish consumption	South Korea	Cross-sectional, stratified random sampling, telephone-based interview, Jul 2012	800	Household member responsible for food purchasing/cooking	20-69	Descriptive statistics, ordered Probit model	Live fish
[85]	Poti et al.	2021	Drivers and barriers to fish consumption	Malaysia	Case study, opportunistic, semi-structured interview, Jul-Sep 2017	73	Household member responsible for buying fish	20-80	Binary logistic regression	Sea turtle egg
[86]	Rahman and Islam	2020	Consumers' preferences for fish attributes	Bangladesh	Cross-sectional, simple random, self-administered, Dec 2019-Jan 2020	128	All types of consumers	n/a	Linear regression	Fish in general
[87]	Sajeev et al.	2021	Drivers and barriers to fish consumption	India	Cross-sectional, stratified proportional, face-to-face interview, Dec 2019-Feb 2020	200	Household member responsible for purchasing food	n/a	Cross-tabulation, Henry Garret ranking test	Fish in general
[88]		2020	Drivers and barriers to fish consumption	India	Cross-sectional, simple random, self-administered, n/a	n/a	All types of consumers	n/a	Cross-tabulation	Fish in general
[89]	Supartini et al.	2018	Drivers and barriers to fish consumption	Singapore	Cross-sectional survey, stratified random, web-based questionnaire, Jan 2017	600	All types of consumers	20-69	Chi-Square, t-test, multiple logistic regression	Fish in general
[90]	Zhou et al.	2015	Drivers and barriers to shellfish consumption	China	Secondary data analysis – China Health and Nutrition Survey (2000, 2004, 2006)	731, 918 and 1083	Household member responsible for purchasing/cooking food	n/a	Generalised least square regression model	Fish in general
[91]	Yadav et al.	2021	Drivers and barriers to fish consumption	Maldives	Cross-sectional, convenience, face-to-face interview, n/a	100	Household member responsible for purchasing/cooking food	18-80	Two-way ANOVA, generalised linear mixed model	Reef fish and tuna

[92]	Tezzo et al.	2021	Fish consumption practices	Myanmar	Qualitative case study, purposive, semi-structured interview, n/a	13 households, 46 individuals in total	All types of consumers	28-62	Social practices approach + Thematic analysis	Fish in general
[93]	Salim et al.	2020	Drivers and barriers to fish consumption	India	Cross-sectional, purposive and simple random, face-to-face interview Mar-May 2019	355	Fish consumers	25-60	Cross-tabulation	Fish in general
[94]	Gohet et al.	2013	Drivers and barriers to fish consumption	Malaysia	Cross-sectional, purposive, self-administered, 2015-2016	385	All types of consumers	18 – 60 and older	Cross-tabulation, ANOVA, Tukey post hoc	Fish and seafood in general
[95]	Salim	2020	Consumers' preferences for fish attributes	India	Cross-sectional, convenience, self-administered, Jan-Dec 2012	600	Household member responsible for buying fish	35 and younger-60 and older	Cross tabulation, logit model	Fish and seafood in general
[96]	Zhang et al.	2021	Drivers and barriers to shellfish consumption	China	Cross-sectional, stratified, face-to-face interview, 2012-2016	1201	Household member responsible for purchasing/cooking food	44 and younger - 89	Ordered probit model, Tobit model	Fish and seafood in general
[97]	Huang et al.	2020	Drivers and barriers to fish consumption	China	Cross-sectional, probability-proportional-to-size, face-to face interview, Aug 2018	424	Household member responsible for food purchasing/cooking	52.33±12.66	Structural equation modelling	Fish in general
[98]	Zheng et al.	2017	Drivers and barriers to fish consumption	China	Cross-sectional, simple random, face-to-face survey, Jun-Jul 2015	1017	All types of consumers	37.80-13.51	Cross tabulation	Alaska salmon
[99]	Zheng et al.	2018	Drivers and barriers to shellfish consumption	China	Cross-sectional, simple random, face-to-face interview, Jun-Jul 2015	1017	All types of consumers	18-85	Descriptive statistics, ordered logit model	Wild salmon
[100]	Wang et al.	2018	Drivers and barriers to fish consumption	China	Cross-sectional, purposive, web-based questionnaire, Dec 2016	882	Consumed lobster before	18-41 and older	Cross-tabulation, t-test, one way ANOVA, partial least squares regression	Lobster
[101]	Wang and Somogyi	2020	Drivers and barriers to fish consumption	China	Cross-sectional, convenience, web-based questionnaire, May 2018	967	All types of consumers	18-70	Descriptive statistic, linear regression model, factor analysis, two-step cluster analysis	Seafood in general

[102]	Skalerud et al.	2012	Drivers and barriers to fish consumption	China	Cross-sectional, random stratified, telephone interview, NA	1200	Female member of the household	15-69	Multinomial logit analysis	Norwegian salmon
[103]	Meng et al.	2021	Drivers and barriers to fish consumption	China	Convenience, face-to-face interview, simple random, self-administered, Jun 2016	394	Household member responsible for food purchasing/cooking	39.24±12.22	Descriptive statistics and Tobit model	Aquatic products
[104]	Juwitaningtyas et al.	2018	Consumers' preferences for fish attributes	Indonesia	Cross-sectional, convenience, self-administered, n/a	101	Fresh fish consumers	n/a	Importance-performance analysis	Fish in general
[105]	Hu and Yuan	2015	Drivers and barriers to fish consumption	China	Cross-sectional, purposive, self-administered, Dec 2011-Feb 2012	1020	University students	19-30	Cross-tabulation	Fish and seafood in general
[106]	Farradia and Sunarno	2020	Drivers and barriers to fish consumption	Indonesia	Cross-sectional, convenience, self-administered, n/a	107	Consumers who prefer to buy online	n/a	Regression and correlation, ANOVA	Fresh fish
[107]	Fabinyi et al.	2016	Drivers and barriers to fish consumption	China	Cross-sectional, quota, web-based questionnaire, Jan – Mar 2015	300	All types of consumers	18-60	Chi-Square test	Aquatic products
[108]	Fabinyi and Liu	2014	Drivers and barriers to seafood consumption	China	Case study, Convenience, semi-structured interview, Apr – Jul 2012	34	20 seafood restaurants managers or chefs, 7 seafood restaurant consumers, 7 trading sector informants	n/a	Thematic analysis	Seafood in general
[109]	Fabinyi and Liu	2016	Drivers and barriers to seafood consumption	China	Ethnographic study, interviews and observations, 2011-2014	n/a	n/a	n/a	Thematic analysis	Seafood in general
[110]	Newton et al.	2021	Drivers and barriers to fish consumption	China	Cross-sectional, simple random, face-to face interview + web-based questionnaire, n/a	267	All types of consumers	n/a	Cross-tabulation	Fish and seafood in general
[111]	Altintzoglou et al.	2016	Drivers and barriers to fish consumption	Japan	Cross-sectional, convenience, web-based questionnaire, n/a	897	All types of consumers	18-64	Cross-tabulation, correlation, ANOVA tests	Sushi and sashimi

References

1. Béné, C.; Arthur, R.; Norbury, H.; Allison, E.H.; Beveridge, M.; Bush, S.; Campling, L.; Leschen, W.; Little, D.; Squires, D.; et al. Contribution of Fisheries and Aquaculture to Food Security and Poverty Reduction: Assessing the Current Evidence. *World Development* **2016**, *79*, 177–196, https://doi.org/10.1016/j.worlddev.2015.11.007

2. Chuenban, P.; Sawmong, S. Determinants of Thailand's Canned Seafood Industry Product Identity: A Structural Equation Model Analysis. *Asia-Pacific Social Science Review* **2018**, *18*, 34–48.
3. Devadawson, C.; Jayasinghe, C.; Sivakanesan, R. Socio-demographic factors and fish eating trends in eastern community, sri lanka. *J. Exp. Biol. Agric. Sci.* **2015**, *3*, 423–429. https://doi.org/10.18006/2015.3(5).423.429
4. Das, A.; Kumar, N.; Debnath, B.; Barman, D.; Datta, M. Fish Consumers' Behaviour at Selected Fish Markets of Tripura, India. *FISHERY TECHNOLOGY* **2013**, *50*, 185–190.
5. Akter, R.; Thilsted, S.; Hossain, N.; Ishihara, H.; Yagi, N. Fish Is the Preferred Animal-Source Food in the Rural Community of Southern Bangladesh. *SUSTAINABILITY* **2019**, *11*, 5764. https://doi.org/10.3390/su11205764
6. Chokenukul, P.; Sukhabot, S.; Rinthaisong, I. A Causal Relationship Model of Purchasing Behavior of Consumers in Thailand Regarding Processed Fish Products. *Kasetsart Journal of Social Sciences* **2019**, *40*, 366–372, https://doi.org/10.1016/j.kjss.2017.10.006
7. Chuenban, P.; Sornsaruht, P.; Pimdee, P. How Brand Attitude, Brand Quality, and Brand Value Affect Thai Canned Tuna Consumer Brand Loyalty. *Heliyon* **2021**, *7*, https://doi.org/10.1016/j.heliyon.2021.e06301
8. Hossain, A.; Badiuzzaman; Nielsen, M.; Hoque, S.; Roth, E. The Role of Private Companies in Improving Nutrition of Consumer Groups in a Developing Country: A Hedonic Analysis of Tilapia Attributes in Bangladesh. *Journal of Applied Aquaculture* **2021**, https://doi.org/10.1080/10454438.2021.1872463
9. Hossain, A. Quantity or Quality of Fish in a Developing Country: A Hedonic Analysis. *Journal of Applied Aquaculture* **2021**, https://doi.org/10.1080/10454438.2021.1970687
10. Intyas, C.A.; Primyastanto, M. Marketing Mix Development Analysis of Preserved Tongkol (Euthynnes Affinis) on Customer Satisfaction in Malang, East Java, Indonesia. In Proceedings of the IOP Conference Series: Earth and Environmental Science; May 1 2020; Vol. 493, p. 012043.
11. Mitra, S.; Khatun, M.N.; Prodhan, M.M.H.; Khan, M.A. Consumer Preference, Willingness to Pay, and Market Price of Capture and Culture Fish: Do Their Attributes Matter? *Aquaculture* **2021**, *544*, https://doi.org/10.1016/j.aquaculture.2021.737139
12. Tan, R.D.; Pabuayon, I.; Rola, A.; Sumalde, Z.M.; Yorobe, J.M. The Price of Fish Quality Attributes in the Province of Iloilo, Philippines: Estimates from Hedonic Pricing for Milkfish. *Asian Fisheries Science* **2017**, *30*, 101–117.
13. Uddin, M.T.; Rasel, M.H.; Dhar, A.R.; Badiuzzaman, M.; Hoque, M.S. Factors Determining Consumer Preferences for Pangas and Tilapia Fish in Bangladesh: Consumers' Perception and Consumption Habit Perspective. *Journal of Aquatic Food Product Technology* **2019**, *28*, 438–449, https://doi.org/10.1080/10498850.2019.1597004
14. Wang, O.; Somogyi, S.; Ablett, R. The Influences of Quality Attributes and Socio-Demographics on Chinese Consumers' General and Online Consumptions of Canadian, US and Australian Lobsters. *British Food Journal* **2021**, *123*, 2289–2306, https://doi.org/10.1108/BFJ-12-2020-1101
15. Wang, O.; Somogyi, S. Chinese Consumers and Shellfish: Associations between Perception, Quality, Attitude and Consumption. *Food Quality and Preference* **2018**, *66*, 52–63, https://doi.org/10.1016/j.foodqual.2018.01.001
16. Ahmed, A.F.; Mohamed, Z.; Ismail, M.M. Determinants of Fresh Fish Purchasing Behavior Among Malaysian Consumers. *Current Research Journal of Social Sciences* **2011**, *3*, 126–131.
17. Omar, M.; Mohd Adzahan, N.; Mohd Ghazali, H.; Karim, R.; Abdul Halim, N.M.; Ab Karim, S. Sustaining Traditional Food: Consumers' Perceptions on Physical Characteristics of Keropok Lekor or Fish Snack. *International Food Research Journal* **2011**, *18*, 117–124.
18. Sajeev, M.; Joshy, C.; Rejula, K.; Sajesh, V.; Suresh, A.; Mohanty, A.; Ravishankar, C. Drivers and Barriers to Online Fish Purchase in Kerala, India. *FISHERY TECHNOLOGY* **2021**, *58*, 246–256.
19. Wijayanto, D.; Bambang, A.N.; Wibowo, B.A.; Mudzakir, A.K. The Characteristics of Demand for Fresh Shrimp in Semarang City, Indonesia. *AACL Bioflux* **2021**, *14*, 1561–1569.
20. Linh, L.T.; Tam, L.M.; Ai, V.D.; Dzung, N.H. ASSESSING CONSUMER BEHAVIOR TOWARDS FISH SAUCE PRODUCTS BY USING FOOD CHOICE QUESTIONNAIRE. *JST* **2019**, *57*, 87, https://doi.org/10.15625/2525-2518/57/3B/14411
21. Castro, M.; Digal, L.; Bayogan, R.; Alcomendras, D. Consumer Preferences for Canned Tuna Products: The Case of the Largest Tuna-Producing City in the Philippines. *AFS* **2016**, *29*, https://doi.org/10.33997/j.afs.2016.29.4.003

22. Izzhati, D.N.; Mastrisiswadi, H.; Jazuli, J.; Talitha, T. Consumer Preferences for The Design of Smoked Fish Packaging Using Conjoint Analysis. *JITI* **2018**, *17*, 177, https://doi.org/10.23917/jiti.v17i2.6778
23. Rejula, K.; Sajeev, M.; Mohanty, A. Health Benefits, Quality and Safety of Fish in Kerala: Consumer Perception and Implications for Extension System. *IJEE* **2021**, *57*, https://doi.org/10.48165/IJEE.2021.57301
24. Mugaonkar, P.H.; Ananthan, P.S.; Samal, S.S.; Debnath, B. A Study on Consumer Behaviour at Organized Fish Retail Outlet. *Agricultural Economics Research Review* **2011**, *24*, 133–140.
25. Zaeema, A.; Hassan, Z. Factors Affecting Purchase Decision of Canned Tuna Brands in Maldives. *IJABM* **2016**, *4*, 12–144, https://doi.org/10.24924/ijabm/2016.04/v4.iss1/120.144
26. Sajiki, T.; Lu, Y.-H. Japanese Consumer Preference For Raw Fish: Best–Worst Scaling Method. *Journal of Marine Science and Technology* **2022**, *29*, 808–816, https://doi.org/10.51400/2709-6998.2560
27. Kitano, S.; Yamamoto, N. The Role of Consumer Knowledge, Experience,and Heterogeneity in Fish Consumption: Policy Lessons from Japan. *Journal of Retailing and Consumer Services* **2020**, *56*, https://doi.org/10.1016/j.jretconser.2020.102151
28. Mugaonkar, P.H.; Kumar, N.R.; Krishnan, M. Seafood Labelling: Awareness and Perception among Consumers of Organized Fish Retail Outlets in India. *Indian Journal of Marketing* **2013**, *43*, 5–13, https://doi.org/10.17010/ijom/2013/v43/i11/80502
29. Alam, M.A.; Alfnes, F. Consumer Preferences for Fish Attributes in Bangladesh: A Choice Experiment. *Journal of International Food and Agribusiness Marketing* **2020**, *32*, 425–440, https://doi.org/10.1080/08974438.2019.1697409
30. Ariji, M. Conjoint Analysis of Consumer Preference for Bluefin Tuna. *Fisheries Science* **2010**, *76*, 1023–1028, https://doi.org/10.1007/s12562-010-0297-4
31. Hoque, M.Z. Sustainability Indicators for Sustainably-Farmed Fish in Bangladesh. *Sustainable Production and Consumption* **2021**, *27*, 115–127, https://doi.org/10.1016/j.spc.2020.10.020
32. Hoque, M.Z.; Akhter, N.; Mawa, Z. Consumers' Willingness to Pay (WTP) for Organically Farmed Fish in Bangladesh. *J. Agric. Appl. Econ.* **2021**, *53*, 482–509, https://doi.org/10.1017/aae.2021.12
33. Hoque, M.Z.; Alam, M.N. Consumers' Knowledge Discrepancy and Confusion in Intent to Purchase Farmed Fish. *British Food Journal* **2020**, *122*, 3567–3583, https://doi.org/10.1108/BFJ-01-2019-0021
34. Hoque, M.Z.; Bashar, M.A.; Akhter, F. Ambiguity Tolerance and Confusion Avoidance in the Intent to Purchase Farmed Fish. *Journal of Applied Aquaculture* **2021**, https://doi.org/10.1080/10454438.2021.1872462
35. Hori, J.; Wakamatsu, H.; Miyata, T.; Oozeki, Y. Has the Consumers Awareness of Sustainable Seafood Been Growing in Japan? Implications for Promoting Sustainable Consumerism at the Tokyo 2020 Olympics and Paralympics. *Marine Policy* **2020**, *115*, https://doi.org/10.1016/j.marpol.2020.103851
36. Karnad, D.; Gangadharan, D.; Krishna, Y.C. Rethinking Sustainability: From Seafood Consumption to Seafood Commons. *Geoforum* **2021**, *126*, 26–36, https://doi.org/10.1016/j.geoforum.2021.07.019
37. Kim, B.-T.; Lee, M.-K. Consumer Preference for Eco-Labeled Seafood in Korea. *Sustainability* **2018**, *10*, 3276, https://doi.org/10.3390/su10093276
38. Murakami, M.; Suzuki, M.; Yamaguchi, T. Presenting Information on Regulation Values Improves the Public's Sense of Safety: Perceived Mercury Risk in Fish and Shellfish and Its Effects on Consumption Intention. *PLoS ONE* **2017**, *12*, https://doi.org/10.1371/journal.pone.0188758
39. Prince, S.A.; Saira Wahid, I. The Purchase of Organic Fish in Bangladesh: Safeguarding against COVID-19. *Cogent Business and Management* **2020**, *7*, https://doi.org/10.1080/23311975.2020.1841524
40. Zheng, Q.; Wang, H.H.; Shogren, J.F. Fishing or Aquaculture? Chinese Consumers' Stated Preference for the Growing Environment of Salmon through a Choice Experiment and the Consequentiality Effect. *Marine Resource Economics* **2021**, *36*, 23–41, https://doi.org/10.1086/711385
41. Omar, N.A.; Abdullah, N.L.; Zainol, Z.; Nazri, M.A. Consumers' Responsiveness towards Contaminated Canned Sardine in Malaysia: Does Perceived Severity Matter? *Food Control* **2021**, *123*, https://doi.org/10.1016/j.foodcont.2020.107780
42. Sayeed, Z.; Sugino, H.; Sakai, Y.; Yagi, N. Consumer Preferences and Willingness to Pay for Mud Crabs in Southeast Asian Countries: A Discrete Choice Experiment. *Foods* **2021**, *10*, https://doi.org/10.3390/foods10112873
43. Tan, R.D. Comparing Awareness and Behaviour Towards Food Consumption Trends: Gender Differences among Milkfish, Chanos Chanos (Forsskål 1775), Purchase Decision Makers in the Province of Iloilo, Philippines. *AFS* **2017**, *30S*, 291-301. https://doi.org/10.33997/j.afs.2017.30.S1.016

44. Uchida, H.; Onozaka, Y.; Morita, T.; Managi, S. Demand for Ecolabeled Seafood in the Japanese Market: A Conjoint Analysis of the Impact of Information and Interaction with Other Labels. *Food Policy* **2014**, *44*, 68–76, https://doi.org/10.1016/j.foodpol.2013.10.002
45. Wakamatsu, H.; Anderson, C.M.; Uchida, H.; Roheim, C.A. Pricing Ecolabeled Seafood Products with Heterogeneous Preferences: An Auction Experiment in Japan. *Marine Resource Economics* **2017**, *32*, 277–294, https://doi.org/10.1086/692029
46. Wakamatsu, H.; Miyata, T. Reputational Damage and the Fukushima Disaster: An Analysis of Seafood in Japan. *Fisheries Science* **2017**, *83*, 1049–1057, https://doi.org/10.1007/s12562-017-1129-6
47. Xu, P.; Zeng, Y.; Fong, Q.; Lone, T.; Liu, Y. Chinese Consumers' Willingness to Pay for Green- and Eco-Labeled Seafood. *Food Control* **2012**, *28*, 74–82, https://doi.org/10.1016/j.foodcont.2012.04.008
48. Xuan, B.; Sandorf, E.; Ngoc, Q. Stakeholder Perceptions towards Sustainable Shrimp Aquaculture in Vietnam. *JOURNAL OF ENVIRONMENTAL MANAGEMENT* **2021**, *290*, https://doi.org/10.1016/j.jenvman.2021.112585
49. Xuan, B.B. Consumer Preference for Eco-Labelled Aquaculture Products in Vietnam. *Aquaculture* **2021**, *532*, https://doi.org/10.1016/j.aquaculture.2020.736111
50. Yi, S. Determinants of Consumers' Purchasing Behavior for Certified Aquaculture Products in South Korea. *Sustainability* **2019**, *11*, 3840, https://doi.org/10.3390/su11143840
51. Yi, S. Willingness to Pay for Sustainable Aquaculture Products: Evidence from Korean Red Seabream Aquaculture. *Sustainability* **2019**, *11*, 1577, https://doi.org/10.3390/su11061577
52. Yin, S.; Han, F.; Chen, M.; Li, K.; Li, Q. Chinese Urban Consumers' Preferences for White Shrimp: Interactions between Organic Labels and Traceable Information. *Aquaculture* **2020**, *521*, https://doi.org/10.1016/j.aquaculture.2020.735047
53. Zhang, X.; Fang, Y.; Gao, Z. Accounting for Attribute Non-Attendance (ANA) in Chinese Consumers' Away-from-Home Sustainable Salmon Consumption. *Marine Resource Economics* **2020**, *35*, 263–284, https://doi.org/10.1086/709458
54. Peng, J.; Min, S.; Qing, P.; Yang, M. The Impacts of Urbanization and Dietary Knowledge on Seaweed Consumption in China. *Foods* **2021**, *10*, 1373, https://doi.org/10.3390/foods10061373
55. Ghosh, A.; Dana, S.; Sharma, A.; Das, S. A Study on Consumers Preference and Satisfaction towards Ready-to-Eat Fish Products in West Bengal, India. *FISHERY TECHNOLOGY* **2018**, *55*, 282–287.
56. Abidin, Z.; Setiawan, B.; Muhaimin, A.W.; Shinta, A.; Nandayani, D.R. Millennial Consumer Behavior on Purchasing of Fishery Functional Food at Tokopedia: Testing the Theory of Planned Behavior. In Proceedings of the IOP Conference Series: Earth and Environmental Science; March 1 2021; Vol. 718, p. 012073.
57. Chen, T.; Wang, H. Consumers' Purchase Intention of Wild Freshwater Fish in China from the Perspective of Ecological Protection. *British Food Journal* **2021**, 124, 10, 3308-3322. https://doi.org/10.1108/BFJ-05-2021-0596
58. Dai, Y.; Yuan, Y.-M.; Yuan, Y.; Zhou, Z.; Zhang, H. Factors Influencing Chinese Consumer Attitudes on the Safety of Aquatic Products. *Journal of the World Aquaculture Society* **2021**, 53, 47-59. https://doi.org/10.1111/jwas.12823
59. Fiandari, Y.R.; Surachman, S.; Rohman, F.; Hussein, A.S. Perceived Value Dimension in Repetitive Fish Consumption in Indonesia by Using an Extended Theory of Planned Behavior. *British Food Journal* **2019**, *121*, 1220–1235, https://doi.org/10.1108/BFJ-07-2018-0429
60. Gajaria, T.K.; Mantri, V.A. Perspectives and Attitudes towards the Functional and Safety Aspects of Seaweeds for Edible Applications in India. *Foods* **2021**, *10*, https://doi.org/10.3390/foods10123026
61. Ghifarini, A.; Sumarwan, U.; Najib, M. APPLICATION OF THEORY OF PLANNED BEHAVIOR IN SHRIMP CONSUMER BEHAVIOR ANALYSIS. *INDEPENDENT JOURNAL OF MANAGEMENT & PRODUCTION* **2018**, *9*, 984–1001, https://doi.org/10.14807/ijmp.v9i3.705
62. Li, Y.; Zhong, C. Factors Driving Consumption Behavior for Green Aquatic Products: Empirical Research from Ningbo, China. *British Food Journal* **2017**, *119*, 1442–1458, https://doi.org/10.1108/BFJ-10-2016-0456
63. Novita; Rowena, J. Determinant Factors of Indonesian People's Fish Purchase Intention. *British Food Journal* **2019**, *123*, 2272–2277, https://doi.org/10.1108/BFJ-01-2019-0067
64. Pethiyagoda, N.; Olsen, S. Explaining Fish Consumption Behavior In Sri-Lanka: The Role Of Consideration Set Size, Attitude, Knowledge, Con-Venience Orientation, Price Consciousness, And Variety Seeking Tendency. *Trop Agric Res & Ext* **2013**, *15*, 19, https://doi.org/10.4038/tare.v15i3.5252

65. Quan, S.; Zeng, Y.; Huang, B.; Yabe, M. Chinese Consumers' Purchase Intention of Eco–Labeled Fish : Based on a Survey to Consumers in Beijing. *Journal of the Faculty of Agriculture, Kyushu University* **2014**, *59*, 205–213, https://doi.org/10.5109/1434412
66. Siddique, M.A.M. Explaining the Role of Perceived Risk, Knowledge, Price, and Cost in Dry Fish Consumption Within the Theory of Planned Behavior. *Journal of Global Marketing* **2012**, *25*, 181–201, https://doi.org/10.1080/08911762.2012.743203
67. Thong, N.T.; Olsen, S.O. Attitude toward and Consumption of Fish in Vietnam. *Journal of Food Products Marketing* **2012**, *18*, 79–95, https://doi.org/10.1080/10454446.2012.653778
68. Wang, O.; Somogyi, S. Consumer Adoption of Sustainable Shellfish in China: Effects of Psychological Factors and Segmentation. *Journal of Cleaner Production* **2019**, *206*, 966–975, https://doi.org/10.1016/j.jclepro.2018.09.189
69. Fauziyah, N.S. The Influence of Parents and Other Factors on Adolescents' Fish Consumption in Selected Senior High School Students, Jakarta. *IJPHRD* **2020**, https://doi.org/10.37506/ijphrd.v11i5.9442
70. Junaidi, J.; Ilona, D.; Zaitul, Z. Fresh Fish Consumption Behaviour in Padang City, Indonesia. *ECSOFiM* **2020**, *007*, 197–211, https://doi.org/10.21776/ub.ecsofim.2020.007.02.06
71. Ho Huy, T.; Anh, N.T.; Svein, O.; Vassdal, T. Explaining Repurchase Intention towards Fish: the Extension of the theory of Planned Behavior; International Institute of Fisheries Economics and Trade. Oregon, USA, 2013.
72. Duy, B.T.; Ai, T.H. PURCHASE BEHAVIOR OF ORGANIC SEAFOOD CONSUMERS IN VIETNAM. *EUrASEANs* **2019**, 51–60, https://doi.org/10.35678/2539-5645.1(14).2019.51-60
73. Rotuauli, T.F.; Handaka, A.A.; Rizal, A.; Nurhayati, A. Consumer Behavior Analysis in Purchase of Freshwater Fish in Bandung City (Case Study in the Traditional Market of Kosambi). *AJFAR* **2020**, 1–8, https://doi.org/10.9734/ajfar/2020/v6i330096
74. Mugaonkar, P.; Kumar, N.; Shelar, G.; Polanco, J.; Ramsubramanian, V.; Biradar, R. A Case Study on the Non-Price Factors and Consumer Behaviour for Pangasius (Pangasianodon Hypophthalmus) (Valenciennes, 1840) Consumption in Pune City, India. *FISHERY TECHNOLOGY* **2017**, *54*, 279–286.
75. Danso, G.; Otoo, M.; Linh, N.; Madurangi, G. Households' Willingness-to-Pay for Fish Product Attributes and Implications for Market Feasibility of Wastewater-Based Aquaculture Businesses in Hanoi, Vietnam. *Resources* **2017**, *6*, 30, https://doi.org/10.3390/resources6030030
76. Ahmad, N.I.; Wan Mahiyuddin, W.R.; Tengku Mohamad, T.R.; Ling, C.Y.; Daud, S.F.; Hussein, N.C.; Abdullah, N.A.; Shaharudin, R.; Sulaiman, L.H. Fish Consumption Pattern among Adults of Different Ethnics in Peninsular Malaysia. *Food & Nutrition Research* **2016**, *60*, 32697, https://doi.org/10.3402/fnr.v60.32697
77. Arthatiani, F.Y.; Luhur, E.S.; Wardono, B.; Yulisti, M. Socio-Economic Determinants of Preserved Fish Consumption in Java Island: SUSENAS Data Analysis 2019. In IOP Conference Series: Earth and Environmental Science, 2021; Vol. 870, 1, 012046. https://doi.org/10.1088/1755-1315/870/1/012046
78. Devadawson, C.; Jayasinghe, C.; Sivakanesan, R. SOCIO-DEMOGRAPHIC FACTORS AND FISH EATING TRENDS IN EASTERN COMMUNITY, SRI LANKA. *JOURNAL OF EXPERIMENTAL BIOLOGY AND AGRICULTURAL SCIENCES* **2015**, *3*, 423–429, https://doi.org/10.18006/2015.3(5).423.429
79. Fu, Y.; Huang, S.; Wu, Z.; Wang, C.; Su, M.; Wang, X.; Xu, P.; Huang, X.; Wu, H.; Wang, Y.; et al. Socio-demographic Drivers and Public Perceptions of Consumption and Conservation of Asian Horseshoe Crabs in Northern Beibu Gulf, China. *Aquatic Conserv: Mar Freshw Ecosyst* **2019**, *29*, 1268–1277, https://doi.org/10.1002/aqc.3125
80. Haque, M.E.; Khanom, S.; Afrad, M.S.I.; Barau, A.A.; Rafiquzzaman, S. Consumer Preference for Sea Fish Consumption in Dhaka City of Bangladesh. *Agriculturists* **2019**, *17*, 41–51, https://doi.org/10.3329/agric.v17i1-2.44695
81. Hoa, V.T.; Käre, S. Preference-Based Segmentation: A Study of Dish Preferences among Vietnamese Teenagers. *Journal of Economic Development* **2017**, *24*, 132–153, https://doi.org/10.24311/JABES/2017.24.2.02
82. Islam, M.J.; Sayeed, M.A.; Akhtar, S.; Hossain, M.S.; Liza, A.A. Consumers Profile Analysis towards Chicken, Beef, Mutton, Fish and Egg Consumption in Bangladesh. *British Food Journal* **2018**, *120*, 2818–2831, https://doi.org/10.1108/BFJ-03-2018-0191
83. Kashem, Md.A.; Tasnim, N.; Rahman, M.; Bapary, M.A.J.; Akanda, Md.A. Consumer's Attitudes toward Fish Consumption during Pandemic Covid-19 in Bangladesh. *International Journal of Natural Sciences* **2021**, *11*, 15–22.

84. Lee, M.-K.; Nam, J. The Determinants of Live Fish Consumption Frequency in South Korea. *Food Research International* **2019**, *120*, 382–388, https://doi.org/10.1016/j.foodres.2019.03.005
85. Poti, M.; Long, S.L.; Rusli, M.U.; Jani, J.M.; Hugé, J.; Dahdouh-Guebas, F. Changing Trends and Perceptions of Sea Turtle Egg Consumption in Redang Island, Malaysia. *Ecology and Society* **2021**, *26*, https://doi.org/10.5751/ES-12717-260414
86. Rahman, M.N.; Islam, A.R.M.T. Consumer Fish Consumption Preferences and Contributing Factors: Empirical Evidence from Rangpur City Corporation, Bangladesh. *Heliyon* **2020**, *6*, https://doi.org/10.1016/j.heliyon.2020.e05864
87. Sajeev, M.V.; Radhakrishnan, A.; Mohanty, A.K.; Joshy, C.G.; Akber Ali, V.P.; Gopika, R.; Mathew, S.; Ravishankar, C.N. Factors Influencing the Fish Consumption Preferences: Understandings from the Tribes of Wayanad, Kerala. *IJEE* **2021**, *57*, 23–27, https://doi.org/10.48165/IJEE.2021.57405
88. Shashikanth H, M.; Somashekar, D. Survey of Fish Consumption Pattern in Households of Shivamogga, Karnataka. *Glob J Zool* **2020**, 022–024, https://doi.org/10.17352/gjz.000016
89. Supartini, A.; Oishi, T.; Yagi, N. Changes in Fish Consumption Desire and Its Factors: A Comparison between the United Kingdom and Singapore. *Foods* **2018**, *7*, https://doi.org/10.3390/foods7070097
90. Zhou, L.; Jin, S.; Zhang, B.; Cheng, G.; Zeng, Q.; Wang, D. Determinants of Fish Consumption by Household Type in China. *British Food Journal* **2015**, *117*, 1273–1288, https://doi.org/10.1108/BFJ-05-2014-0182
91. Yadav, S.; Fisam, A.; Dacks, R.; Madin, J.S.; Mawyer, A. Shifting Fish Consumption Preferences Can Impact Coral Reef Resilience in the Maldives: A Case Study. *Marine Policy* **2021**, *134*, https://doi.org/10.1016/j.marpol.2021.104773
92. Tezzo, X.; Aung, H.M.; Belton, B.; Oosterveer, P.; Bush, S.R. Consumption Practices in Transition: Rural-Urban Migration and the Food Fish System in Myanmar. *Geoforum* **2021**, *127*, 33–45, https://doi.org/10.1016/j.geoforum.2021.09.013
93. Salim, S.S.; Monolisha, S.; Sunil, P.V. Fish Consumption: Gauging the Determinants of Consumption and Buying Patterns across Kerala Markets. *J. Mar. Biol. Ass. India* **2020**, *62*, 33–40, https://doi.org/10.6024/jmbai.2020.62.1.2114-03
94. Goh, E.V.; Ali, S.A.; Mitra, S.R.; McCullough, F. Understanding the Patterns of Fish and Seafood Consumption and Its Nutritional Roles among a Malaysian Population to Inform Sustainable Development. *apjsafe* **2013**, *9*, 1–13, https://doi.org/10.36782/apjsafe.v9i1.86
95. Salim, S.S. Demand Pattern and Willingness to Pay for High Value Fish Consumption: Case Study from Selected Coastal Cities in Kerala, South India. *Indian Journal of Fisheries* **2020**, *67*, 135–143, https://doi.org/10.21077/ijf.2020.67.3.70635-15
96. Zhang, H.; Sun, C.; Wang, Z.; Che, B. Seafood Consumption Patterns and Affecting Factors in Urban China: A Field Survey from Six Cities. *Aquaculture Reports* **2021**, *19*, https://doi.org/10.1016/j.aqrep.2021.100608
97. Huang, J.; Antonides, G.; Nie, F. Social-Psychological Factors in Food Consumption of Rural Residents: The Role of Perceived Need and Habit within the Theory of Planned Behavior. *Nutrients* **2020**, *12*, https://doi.org/10.3390/nu12041203
98. Zheng, Q.; Weng, H.; Fong, Q.; Lu, Y. *Consumer Preference and Market Potential for Alaska Salmon in China: Preliminary Analysis*; Alaska Sea Grant, University of Alaska Fairbanks, 2017;
99. Zheng, Q.; Wang, H.H.; Lu, Y. Consumer Purchase Intentions for Sustainable Wild Salmon in the Chinese Market and Implications for Agribusiness Decisions. *Sustainability (Switzerland)* **2018**, *10*, https://doi.org/10.3390/su10051377
100. Wang, O.; Somogyi, S.; Ablett, R. General Image, Perceptions and Consumer Segments of Luxury Seafood in China: A Case Study for Lobster. *British Food Journal* **2018**, *120*, 969–983, https://doi.org/10.1108/BFJ-07-2017-0379
101. Wang, O.; Somogyi, S. Motives for Luxury Seafood Consumption in First-Tier Cities in China. *Food Quality and Preference* **2020**, *79*, https://doi.org/10.1016/j.foodqual.2019.103780
102. Skallerud, K.; Myrland, Ø.; Olsen, S.O. Buying Norwegian Salmon: A Cross-Cultural Study of Store Choice Behavior. *Journal of Food Products Marketing* **2012**, *18*, 257–267, https://doi.org/10.1080/10454446.2012.684328
103. Meng, T.; Wang, C.; Florkowski, W.J.; Yang, Z. Determinants of Urban Consumer Expenditure on Aquatic Products in Shanghai, China. *Aquaculture Economics and Management* **2021**, 12, 1-24. https://doi.org/10.1080/13657305.2021.1996480

104. Juwitaningtyas, T.; Supartono, W.; Aziz, I.W.F. Consumer's Valuation of the Traditional Fish Handling Practices Using Importance-Performance Analysis. In Proceedings of the IOP Conference Series: Materials Science and Engineering; October 9 2018; Vol. 403, p. 012008.
105. Hu, Y.; Yuan, C. An Online Survey Study of Consumer Preferences on Aquatic Products in China: Current Seafood Consumption Patterns and Trends. *Fish Aquac J* **2015**, *05*, https://doi.org/10.4172/2150-3508.1000094
106. Farradia, Y.; Sunarno, M.T.D. Consumer Purchase Decision on Fresh Fish in the New Norm: Preliminary Case Study in Indonesia. *JSTARD* **2020**, *2*, https://doi.org/10.30880/jstard.2020.02.01.006
107. Fabinyi, M.; Liu, N.; Song, Q.; Li, R. Aquatic Product Consumption Patterns and Perceptions among the Chinese Middle Class. *Regional Studies in Marine Science* **2016**, *7*, 1–9, https://doi.org/10.1016/j.rsma.2016.01.013
108. Fabinyi, M.; Liu, N. Seafood Banquets in Beijing: Consumer Perspectives and Implications for Environmental Sustainability. *Conservation and Society* **2014**, *12*, 218–228, https://doi.org/10.4103/0972-4923.138423
109. Fabinyi, M.; Liu, N. The Social Context of the Chinese Food System: An Ethnographic Study of the Beijing Seafood Market. *Sustainability (Switzerland)* **2016**, *8*, https://doi.org/10.3390/su8030244
110. Newton, R.; Zhang, W.; Xian, Z.; McAdam, B.; Little, D.C. Intensification, Regulation and Diversification: The Changing Face of Inland Aquaculture in China. *Ambio* **2021**, *50*, 1739–1756, https://doi.org/10.1007/s13280-021-01503-3
111. Altintzoglou, T.; Heide, M.; Wien, A.H.; Honkanen, P. Traditional Sushi for Modern Consumers: A Comparison Between Sushi Consumption Behavior in Japan and Norway. *Journal of Food Products Marketing* **2016**, *22*, 717–732, https://doi.org/10.1080/10454446.2015.1121434
